# Early infiltrating NKT lymphocytes attenuate bone regeneration through secretion of CXCL2

**DOI:** 10.1126/sciadv.adl6343

**Published:** 2024-05-17

**Authors:** Weimin Lin, Qiwen Li, Linfeng Liu, Qian Wang, Danting Zhang, Feiyu Wang, Ruoshi Xu, Yi Fan, Malcolm Xing, Chenchen Zhou, Quan Yuan

**Affiliations:** ^1^ State Key Laboratory of Oral Diseases and National Center for Stomatology & National Clinical Research Center for Oral Diseases, West China Hospital of Stomatology, Sichuan University, 610041 Chengdu, Sichuan, China.; ^2^ Department of Cariology and Endodontics, West China Hospital of Stomatology, Sichuan University, 610041 Chengdu, Sichuan, China.; ^3^ Department of Mechanical Engineering, University of Manitoba, Winnipeg R3T 2N2, Canada.; ^4^ Department of Pediatric Dentistry, West China Hospital of Stomatology, Sichuan University, 610041 Chengdu, Sichuan, China.; ^5^ Department of Oral Implantology, West China Hospital of Stomatology, Sichuan University, 610041 Chengdu, Sichuan, China.

## Abstract

Trauma rapidly mobilizes the immune response of surrounding tissues and activates regeneration program. Manipulating immune response to promote tissue regeneration shows a broad application prospect. However, the understanding of bone healing dynamics at cellular level remains limited. Here, we characterize the landscape of immune cells after alveolar bone injury and reveal a pivotal role of infiltrating natural killer T (NKT) cells. We observe a rapid increase in NKT cells after injury, which inhibit osteogenic differentiation of mesenchymal stem cells (MSCs) and impair alveolar bone healing. *Cxcl2* is up-regulated in NKT cells after injury. Systemic administration of CXCL2-neutralizing antibody or genetic deletion of *Cxcl2* improves the bone healing process. In addition, we fabricate a gelatin-based porous hydrogel to deliver NK1.1 depletion antibody, which successfully promotes alveolar bone healing. In summary, our study highlights the importance of NKT cells in the early stage of bone healing and provides a potential therapeutic strategy for accelerating bone regeneration.

## Teaser

A cell atlas of bone injury repair is constructed to reveal contribution of NKT cells.

## INTRODUCTION

The bone healing process consists of several sequential and overlapping stages, including hematoma and inflammation phase, repair and regeneration phase, and bone remodeling phase (*1*). After bone tissue injury, pro-inflammatory factors and regeneration-related factors cooperate to regulate angiogenesis and the migration and differentiation of different bone precursor cells to initiate bone regeneration process. The subsequent upregulation of anti-inflammatory factors is beneficial to ensure a balanced inflammatory response process and ideal bone regeneration (*2*, *3*). In the early stage of inflammation, immune cells are quickly recruited to the injury site and secreting various inflammatory factors to initiate the inflammatory process. Under pathological condition, the prolonged inflammatory process exposing the tissue to an overactivated inflammatory environment could impair bone healing (*4*). Therefore, appropriate immune cell population switching is particularly important for bone regeneration during the early inflammatory phase.

Dysregulation of inflammatory response in the early stage of healing impairs normal bone repair process. Most of the current treatment strategies for bone defects are aimed at improving osteogenic differentiation and bone formation in the later stage of bone healing (*5*). In recent years, with the concept of “osteoimmunology,” more attention has been paid to the role of the immune regulation in bone injury (*6*, *7*). However, because a variety of immune cells are involved in the regulation of the healing process in the early stage of acute inflammation, the contribution of specific immune subtypes has not been clearly studied, and the corresponding therapeutic targets remain to be explored.

Single-cell RNA sequencing (scRNA-seq) technology is considered to be an effective tool for studying cellular components and their interactions in the tissue microenvironment (*8*–*10*). However, because of the technical difficulty of preparing high-quality single-cell suspensions from bone tissue, single-cell omics studies in the field of bone regeneration are still limited. The complete transcriptomic atlas of bone marrow stromal cells was firstly constructed through flow sorting technique (*11*, *12*). scRNA-seq analysis of the *Prx1*^+^ cell from muscle tissue near the fracture site proved the direct contribution of skeletal muscle mesenchymal progenitors to fracture healing (*13*). In our previous study, we identified that macrophage population regulated alveolar bone homeostasis through Oncostatin M (OSM) signaling pathway (*14*). So far, systematic studies of the dynamic cellular atlas of immune cells during early bone repair are still lacking.

In the current study, we dissect the osteoimmune microenvironment at five different time points before and after bone injury by scRNA-seq. Our data indicate that natural killer T (NKT) lymphocytes massively infiltrate into the injured site at the early stage. NKT cell–derived CXCL2 is shown to impair alveolar bone healing. Topical delivery of NK1.1 depletion antibody through hydrogel effectively promotes bone repair and avoids potential side effects of systemic administration. Our findings reveal an important regulatory role of NKT cell in early stage of alveolar bone repair.

## RESULTS

### Dynamic atlas of immune cells in alveolar bone healing

**Fig. 1.**
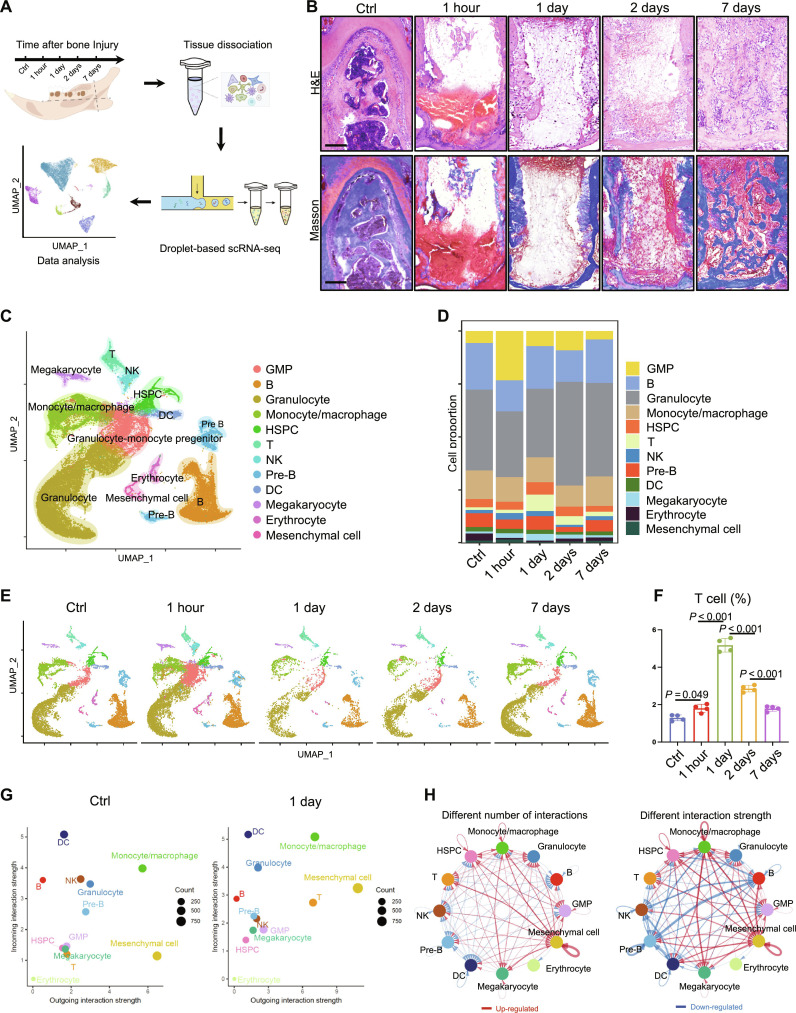
scRNA-seq of alveolar bone after injury. (**A**) Flowchart of single-cell transcriptome study on the alveolar bone of mice after injury at different time points. (**B**) H&E and Masson staining results on the alveolar bone injury site at different time points. Scale bars, 150 μm. (**C**) Combined profiles of all cells at different time points. (**D**) Proportion of different cell types at different time points. (**E**) Uniform Manifold Approximation and Projection (UMAP) plots according to different time points. (**F**) Flow cytometry shows the proportion of CD3E^+^ T lymphocytes at different time points. (**G**) Incoming and outcoming strengths of the cell interaction signals of different cell groups inferred by CellChat package are in the Ctrl group and 1-day group, respectively. (**H**) Differences in intercellular communication between Ctrl group and 1-day group. Red, up-regulated. Blue, down-regulated. GMP, granulocyte-monocyte progenitor; DC, dendritic cell; NK, natural killer; HSPC, hematopoietic stem and progenitor cell.

To investigate the dynamic change of cell populations at different time points after bone injury, we isolated the mouse alveolar bone after teeth extraction and prepared single-cell suspensions for scRNA-seq (Fig. 1A). As for histological characteristics, hematoxylin and eosin (H&E) staining revealed predominantly red blood clots in the alveolar sockets within 1 hour after injury (Fig. 1B). One day after injury, callus formation and infiltrated nucleated cells were present. Seven days after injury, obvious trabecular structures were observed (Fig. 1B). Masson staining confirmed the matured collagen fiber formation on 7 days (Fig. 1B).

After quality control through Cell Ranger software and Seurat package, we obtained 42,072 cells with an average of about 1000 feature_RNAs each cell (fig. S1, A to C). Through the expression pattern of classic cell-type–specific markers, we identified 12 cell populations, including B lymphocyte (*Cd79a*), neutrophil (*Ly6g*), monocyte/macrophage (*Csf1r*), hematopoietic stem and progenitor cell (*Kit*), T lymphocyte (*Cd3e*), granulocyte-monocyte progenitor (*Mpo*), NK cell (*Klrb1c*), pre-B lymphocyte, dendritic cell (*Siglech*), megakaryocyte (*Itga2b*), erythrocyte (*Hbb-bt*), and mesenchymal cell (*Col1a1*) (Fig. 1C and fig. S2, A and B).

Notably, the proportion of T lymphocytes increased almost fourfold (~5.2%) on 1 day, compared to ~1.3% before injury. It decreased steadily from 2 days after injury onward and almost returned to the normal level by 7 days (Fig. 1, D and E). Flow cytometry analysis verified the dynamic changes of CD3E^+^ T cells after bone injury (Fig. 1F and fig. S3).

Further, we compared the intercellular communications between Ctrl group and 1-day group through CellChat package. Both incoming and outgoing interaction strengths of the T cell population were improved compared to Ctrl group (Fig. 1G). Notably, the interaction between T cells and mesenchymal cells was increased on 1 day, suggesting that T cells might play an important role in regulating bone healing process (Fig. 1H).

We further performed subcluster analysis on mesenchymal cells and monocyte/macrophage populations. Within the mesenchymal cell population, six common nonimmune cell subtypes were identified, including endothelial cells (*Cdh5*), mesenchymal stem cells (MSCs; *Lepr* and *Cxcl12*), osteogenic cells (*Sp7* and *Dmp1*), fibroblastic cells (*Col1a1* and *Dcn*), myocytes (*Acta2*), and neurological cells (*Plp1*) (fig. S4, A and B). Pseudo-time analysis suggested that MSCs have bifurcated differentiation trajectories: osteogenic differentiation and fibrogenic differentiation (fig. S4, C and D). Through enrichment analysis of genes related to the differentiation trajectory, we found that MSC-related genes were mainly enriched in pathways such as cell proliferation and migration. Osteoblast-related genes were enriched in bone formation–related pathways. Fibroblast-related genes were enriched in immune response–related pathways, suggesting that during bone injury repair, fibroblasts might be involved in the regulation of the inflammatory response and resistance to infection (fig. S4E). In addition, subcluster analysis of the monocyte/macrophage population indicated that the proportion of M2 macrophages (anti-inflammatory type), marked by *Cd36* and *Cd163*, dropped to the lowest on 1 and 2 days and then recovered to pre-injury levels on 7 days (fig. S5, A and B).

### Dynamic change of T cell subclusters after bone injury

**Fig. 2.**
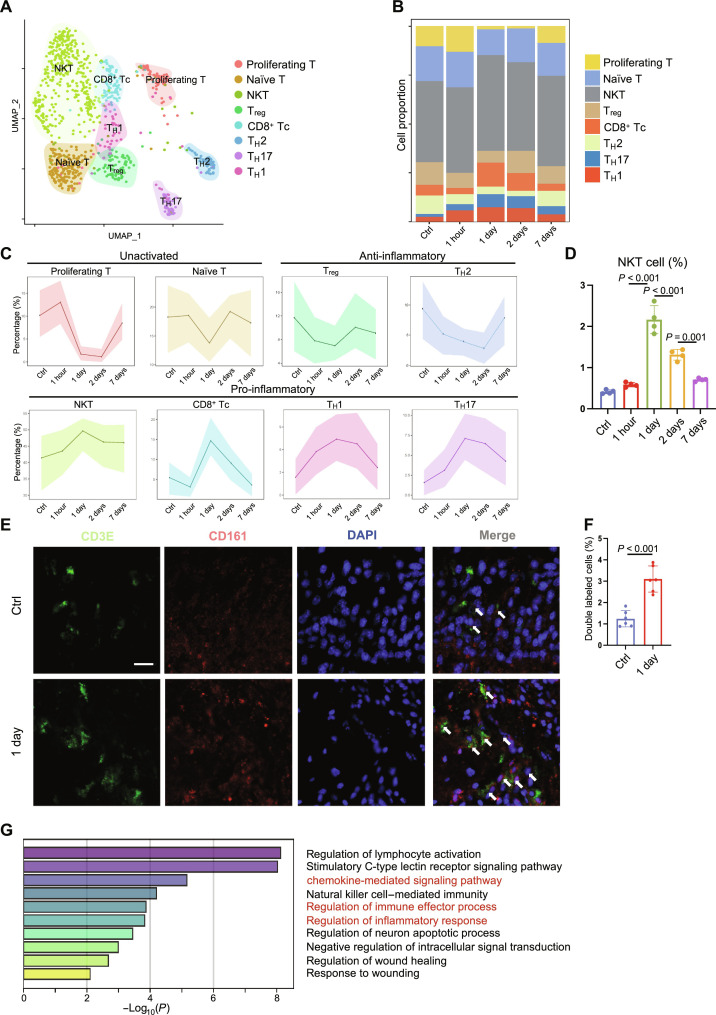
Dynamic changes of T cell subsets at different time points. (**A**) UMAP plot of different T cell subsets after identification. (**B**) Changes in the proportion of each T cell subset at different time points. (**C**) Cell-type proportion inference of T cell subclusters. (**D**) Flow cytometry shows the proportion of CD3E^+^ NK1.1^+^ NKT cells at different time points. (**E**) Immunofluorescence staining of CD3E^+^ and CD161^+^ NKT cells. Scale bar, 50 μm. (**F**) Statistical analysis of CD3E^+^ and CD161^+^ cell proportion in all nucleated cells. (**G**) GO enrichment analysis result of NKT cell–specific genes. DAPI, 4′,6-diamidino-2-phenylindole.

Next, we divided T cells into nine subclusters, including NKT (*Klrb1c*), cytotoxic T cell (Tc; *Ifng*), regulatory T cell (T_reg_; *Foxp3*), T helper cell 1 (T_H_1; *Tbx21*), T_H_2 (*Gata3*), T_H_17 (*Il17*), proliferating T cell (*Mki67*), and naïve T cell (*Ccr7*) (Fig. 2A and fig. S6, A and B). Among them, NKT cells accounted for the highest population (~40%) (Fig. 2B). In NKT cell population, the expression of type 1 cytokines (*Ifng* and *Tnf*) is relatively high, while the expression of type 2 cytokines (*Il4*, *Il5*, *Il10*, and *Il13*) and type 17 cytokines (*Il17a*, *Il17f*, and *Il22*) is almost undetectable (fig. S7). The proportion of NKT cell to all T cells was also the highest on 1 day and restored to a comparable level to Ctrl group on 7 days. The proportions of several T cell subclusters that were considered to be pro-inflammatory, including Tc, T_H_17, and T_H_1 cells, were highest on 1 day and returned to a level similar to the Ctrl group on 7 days. The proportions of T_reg_ cells and T_H_2 cells reached the lowest on 1 day and then recovered (Fig. 2, B and C).

We then performed flow cytometry analysis and verified that the proportion of CD3E^+^ and NK1.1^+^ NKT cells to all cells rose from ~0.5% before injury to the highest level of ~2.4% on 1 day and gradually declined to normal levels on 7 days (Fig. 2D). CD1d tetramer–labeled invariant NKT cells accounted for approximately 40% of CD3E^+^ NK1.1^+^ cells, and their proportion did not change during the healing process (fig. S8, A to C). Immunofluorescence staining of CD3E and CD161 on the bone sections further confirmed this observation (Fig. 2, E and F). Gene Ontology (GO) enrichment analysis showed that NKT cells were associated with chemokine-mediated signaling pathway and inflammatory response (Fig. 2G).

### Increased NKT cells negatively regulate bone repair

**Fig. 3.**
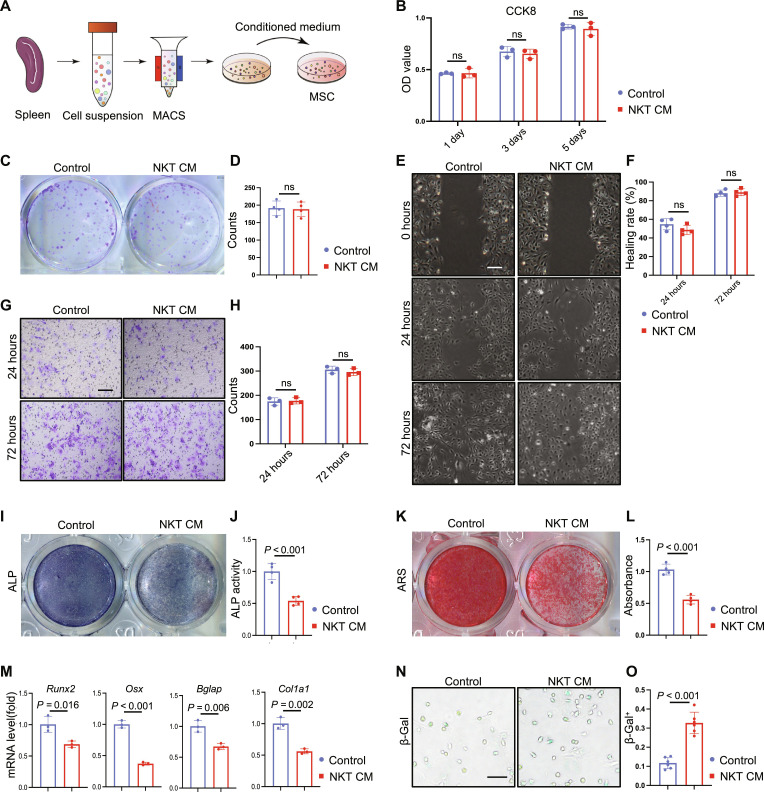
NKT cells inhibit the osteogenic differentiation of MSC in vitro. (**A**) Flow chart of magnetic bead sorting mouse NKT cells. (**B**) MSC proliferation ability analysis and statistics after NKT cell conditioned medium treatment. (**C** and **D**) MSC colony formation analysis and data statistics after NKT CM treatment. (**E** and **F**) Scratch healing assay and data statistics under NKT CM treatment. Scale bar, 50 μm. (**G** and **H**) Transwell assay result of MSC under NKT CM treatment. Scale bar, 50 μm. (**I** and **J**) ALP staining and ALP activity measurement of MSCs after induction for 7 days. (**K** and **L**) ARS staining and semi-quantitative analysis results of MSC after 14 days of induction. (**M**) Reverse transcription quantitative polymerase chain reaction (RT-qPCR) results of osteogenic differentiation-related gene expression levels after 5 days of induction. (**N** and **O**) SA-β-Gal staining of MSC and statistical analysis of positive cells after induction of 3 days.

To explore the role of increased NKT cells on bone repair, we cultured mouse primary NKT cells and collected their conditioned medium to treat MSCs (Fig. 3A). Supplementation of NKT cell–conditioned medium (NKT CM) did not affect the proliferation (Fig. 3B) or clonogenic potential (Fig. 3, C and D) of MSCs. Transwell and scratch assay showed that NKT CM barely affected the migration of MSCs (Fig. 3, E to H). However, NKT CM treatment significantly reduced the intensity of alkaline phosphatase (ALP) staining and ALP activity (Fig. 3, I and J). Alizarin red S (ARS) staining showed a decreased deposition of calcium nodules (Fig. 3, K and L). Moreover, it inhibited the expression of osteogenesis-related genes, including *Runx2*, *Osx*, *Bglap*, and *Col1a1* (Fig. 3M). The proportion of β-galactosidase^+^ (β-Gal^+^) senescent cells also increased significantly after NKT CM treatment (Fig. 3, N and O). These data indicated that NKT cells inhibit osteogenic differentiation of MSCs in a paracrine manner.

**Fig. 4.**
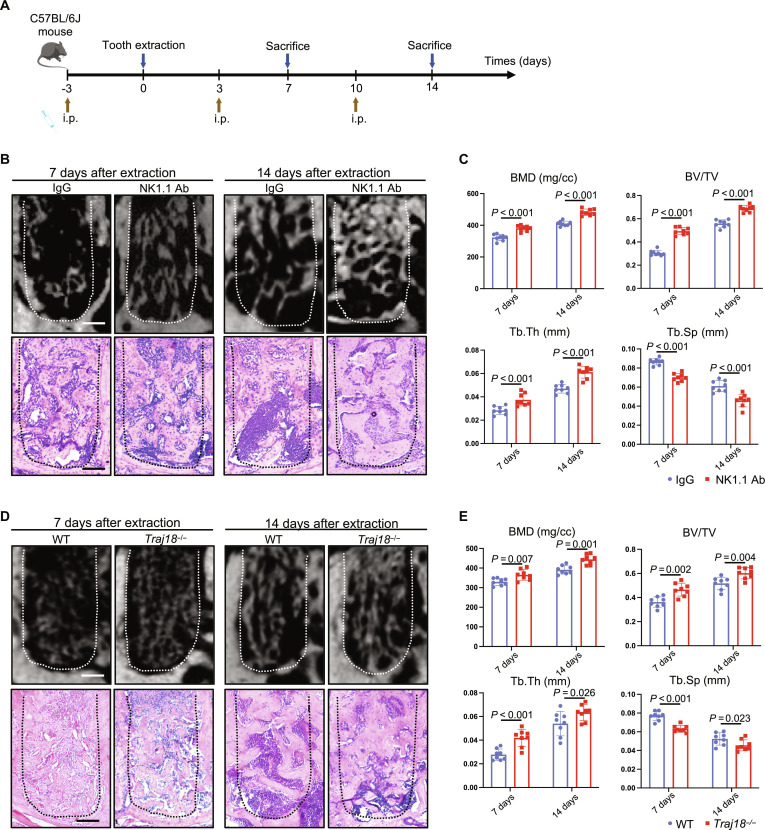
Deletion of NKT cells accelerates alveolar bone healing. (**A**) Flowchart of administration of depletion antibody after alveolar bone injury in mice. (**B**) μCT analysis of alveolar bone injury area at 7 and 14 days and H&E staining after administration of NK1.1 depletion antibody. Scale bars, 150 μm. (**C**) μCT quantitative analysis of regenerated bone volume at the alveolar bone injury site after administration of NK1.1 depletion antibody. (**D**) μCT analysis and H&E staining of alveolar bone injury area at 7 and 14 days in *Traj18*^−/−^ mice. Scale bars, 150 μm. (**E**) μCT quantitative analysis of regenerated bone volume at the alveolar bone injury site in *Traj18*^−/−^ mice. Ab, antibody; IgG, immunoglobulin G; BMD, bone mineral density; BV/TV, bone volume fraction; Tb.Th bone trabecular thickness; Tb.Sp, bone trabecular separation. WT, wild type.

Next, we constructed an in vivo model of NKT cell depletion by injecting NK1.1 depletion antibody (Fig. 4A) (*15*). Flow cytometry demonstrated that the proportion of CD3E^+^ NK1.1^+^ NKT cells significantly decreased from ~0.5 to ~0.03% after injection of NK1.1 depletion antibody (fig. S9). Micro–computed tomography (μCT) analysis showed that injection of NK1.1 antibody increased the bone mineral density (BMD), bone volume fraction (BV/TV), and bone trabecular thickness (Tb.Th) and decreased bone trabecular separation (Tb.Sp). H&E staining of tissue sections verified denser and thicker new bone formation in the NK1.1 antibody–injected mice (Fig. 4, B and C).

As NK1.1 depletion antibody targeting to both NK and NKT cells, we further constructed *Traj18* knockout (*Traj18*^−/−^) mice to exclude the impact of NK cell ablation on bone healing. The deletion of the *Traj18* gene fragment leads to the loss of the T cell receptor α-chain necessary for the development of NKT cells, resulting in the abnormal development of NKT cells (*16*). Likewise, *Traj18^−/−^* mice exhibited enhanced bone healing after injury (Fig. 4, D and E). In addition, μCT analysis of the femur demonstrated that injection of NK1.1 depletion antibody and *Traj18* knockout did not affect bone mass (fig. S10, A to D).

### The secretion of CXCL2 in NKT cells increases after injury

**Fig. 5.**
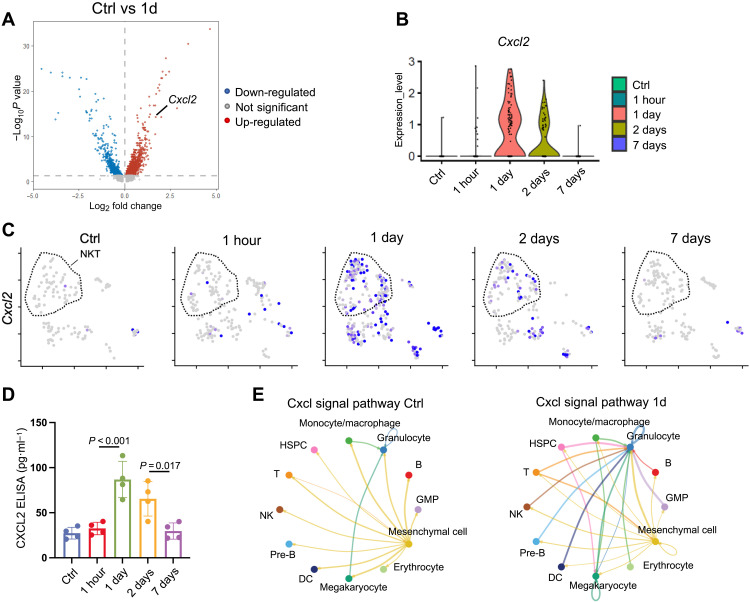
CXCL2 is up-regulated in NKT cells on 1 day. (**A**) Volcano plot shows differentially expressed genes in NKT cell populations in Ctrl and 1-day group. (**B**) Violin plot shows the expression levels of *Cxcl2* in NKT cells at different time points. (**C**) UMAP plot shows the expression pattern of *Cxcl2* in T cell populations at different time points. (**D**) ELISA assay shows the levels of CXCL2 in surrounding tissues after alveolar bone injury. (**E**) Network diagram of *Cxcl2* signal pathway in intercellular communication in Ctrl and 1 day.

To dissect the mechanism how NKT cells hinders bone regeneration, we extracted NKT population and performed differential gene analysis. Seven hundred eighty-six genes were up-regulated, and 1640 genes were down-regulated 1 day after bone injury. Among the top significantly up-regulated genes, we identified *Cxcl2*, an inflammatory factor associated with osteoporosis (*17*) and inflammation development (Fig. 5A) (*18*). In addition, the expression of several other pro-inflammatory cytokines—such as *Ifng*, *Tnfsf9* (encoding 4-1BBL), and *Il1b*—was also up-regulated (fig. S11A). The violin plot and feature plot showed that the expression level of *Cxcl2* was nearly undetectable before injury and at 1 hour, but it increased rapidly on 1 day, slightly decreased on 2 days, and back to normal by 7 days (Fig. 5, B and C). Feature plot also showed that there was a small amount of *Cxcl2* expression in other T cell subsets (Fig. 5C).

To validate the expressional change of *Cxcl2*, we collected alveolar bone at different time points after injury and measured CXCL2 levels by enzyme-linked immunosorbent assay (ELISA). The level of CXCL2 markedly increased and peaked on 1 day and then decreased from 1 to 7 days (Fig. 5D). In addition, the intercellular communication analysis demonstrated that the interaction strength of *Cxcl* pathway was up-regulated between T cells and mesenchymal cells on 1 day (Fig. 5E).

### Elimination of CXCL2 accelerates bone regeneration

**Fig. 6.**
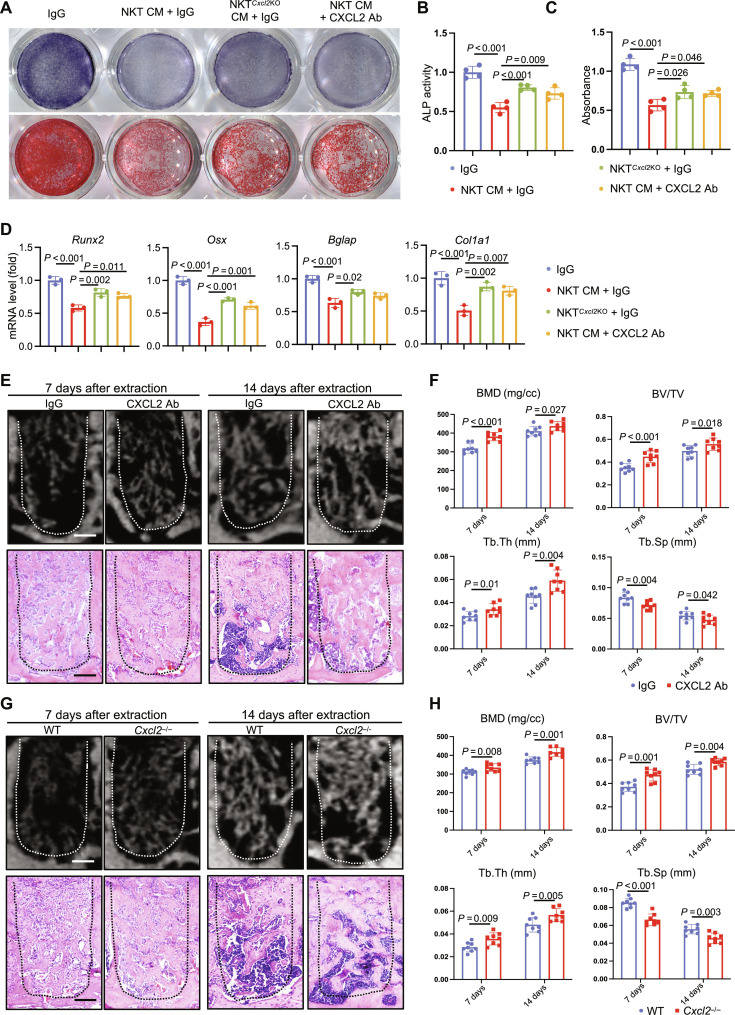
CXCL2 impairs alveolar bone regeneration. (**A**) ALP and ARS staining of MSCs after CXCL2-neutralizing antibody treatment or *Cxcl2* knockout. (**B** and **C**) Quantitative analysis of ALP activity and ARS staining of MSCs after CXCL2-neutralizing antibody treatment or *Cxcl2* knockout. (**D**) Reverse transcription quantitative polymerase chain reaction (RT-qPCR) results of osteogenic differentiation-related genes after CXCL2-neutralizing antibody treatment or *Cxcl2* knockout. (**E**) μCT analysis and H&E staining of alveolar bone injury area at 7 and 14 days after CXCL2-neutralizing antibody treatment. Scale bars, 150 μm. (**F**) μCT quantitative analysis of regenerated bone volume at the alveolar bone injury site after CXCL2-neutralizing antibody treatment. (**G**) μCT analysis and H&E staining of alveolar bone injury area at 7 and 14 days in *Cxcl2*^−/−^ mice. Scale bars, 150 μm. (**H**) μCT quantitative analysis of regenerated bone volume at the alveolar bone injury site in *Cxcl2*^−/−^ mice. Ab, antibody; IgG, immunoglobulin G.

To determine whether the inhibitory effect of NKT on bone repair is mediated by CXCL2, we treated cells with CXCL2-neutralizing antibody. CXCL2 neutralization or *Cxcl2* knockout partially rescued the NKT CM–mediated inhibition of osteogenic differentiation. After administration of CXCL2-neutralizing antibodies or *Cxcl2* knockout, ALP staining intensity of MSCs increased after induction, and ALP activity was also partially rescued (Fig. 6, A and B). ARS staining showed an increase in the area of red-stained calcium nodules. (Fig. 6, A and C). The expressions of osteogenic genes were partially up-regulated (Fig. 6D). Although neutralizing other up-regulated cytokines, including Interferon Gamma (IFNG), Tumor Necrosis Factor Superfamily Member 9 (TNFSF9), Interleukin 1 Beta (IL1B), also ameliorated the osteogenic differentiation to some extent, CXCL2 antibody showed the strongest effect (fig. S11, B to E).

Next, we sought to test the effect in vivo by intraperitoneal injection of CXCL2-neutralizing antibodies. μCT showed more new bone formation in alveolar sockets of CXCL2-neutralizing antibody injection group, and H&E histological staining also proved that the trabecular bone was denser and thicker in the antibody injection group (Fig. 6E). Quantitative analysis also showed that the BMD, BV/TV, and Tb.Th of the CXCL2-neutralizing antibody administration group were significantly higher than those of the Ctrl group, and Tb.Sp was decreased (Fig. 6F).

In addition, we constructed *Cxcl2* knockout (*Cxcl2*^−/−^) mice. *Cxcl2*^−/−^ mice exhibited enhanced bone healing compared to wild-type (WT) control in H&E histological staining. μCT reconstruction and quantitative analysis also demonstrated that the regenerated bone had better quality in *Cxcl2*^−/−^ mice (Fig. 6, G and H). μCT analysis of the femur demonstrated that injection of CXCL2-neutralizing antibody, and *Cxcl2* knockout did not affect bone mass (fig. S12, A to D). Administration of CXCL2-neutralizing antibodies to *Traj18*^−/−^ mice also improved the bone repair process. There were significant differences in BMD, BV/TV, and Tb.Th between the CXCL2-neutralizing antibody group and the *Traj18*^−/−^ group at 7 days (fig. S13, A and B).

### Fabrication of porous GelMA hydrogel for rapid topical release of loaded drug

**Fig. 7.**
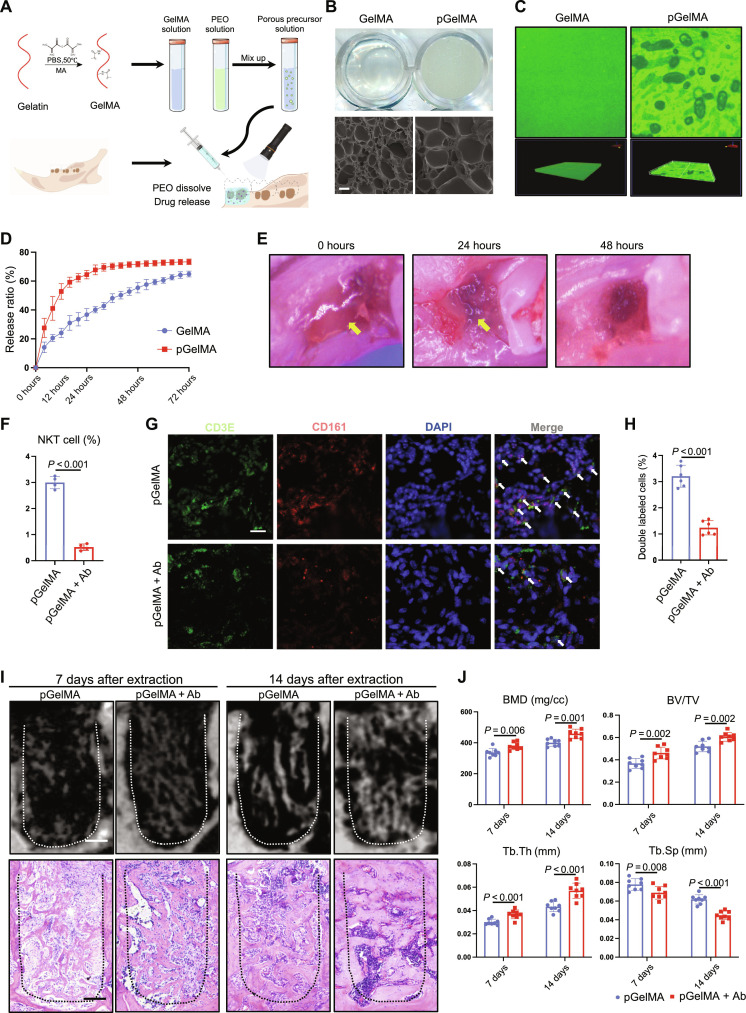
Topical delivery of drugs via hydrogel accelerates bone healing. (**A**) Flowchart of preparation of pGelMA hydrogel and topical delivery of drugs through it at the alveolar bone injury site. (**B**) General view and SEM images of GelMA and pGelMA hydrogels. Scale bar, 10 μm. (**C**) Laser confocal images identify porous structures in hydrogels. (**D**) Drug release rate of GelMA and pGelMA. (**E**) Degradation of pGelMA hydrogel after topical application at the injured site. (**F**) Flow cytometry detection of the proportion of NKT cells after application of drug-loaded pGelMA hydrogel. (**G**) Immunofluorescence staining of CD3E^+^ and CD161^+^ NKT cells after topical application of drug-loaded pGelMA hydrogel. Scale bar, 50 μm. (**H**) Statistical analysis of CD3E^+^ and CD161^+^ cells proportion in all nucleated cells. (**I**) μCT analysis and H&E staining of the alveolar bone injury area at 7 and 14 days after topical application of drug-loaded pGelMA hydrogel. Scale bars, 150 μm. (**J**) μCT quantitative analysis of regenerated bone volume at the alveolar bone injury site after topical application of drug-loaded pGelMA hydrogel.

To avoid risks of global manipulation, we introduced micron-sized pores into the gelatin methylacryloyl (GelMA) hydrogel by phase separation and then loaded the hydrogel with NK1.1 depletion antibody (Fig. 7A). In general view, porous GelMA (pGelMA) hydrogel appeared more opaque than ordinary GelMA hydrogel. Scanning electron microscopy (SEM) showed that pGelMA hydrogel had a larger internal pore structure (Fig. 7B). Laser confocal microscopy scanning showed that the hydrogel prepared by the liquid phase separation method had a typical porous structure (Fig. 7C). The pGelMA hydrogel released more than 60% of loaded drug after 24 hours, whereas the conventional GelMA hydrogel reached that after about 72 hours (Fig. 7D). When injected into the tooth extraction wound and solidified, part of the hydrogel remained at the wound site at 24 hours, and majority of it degraded at 48 hours (Fig. 7E).

Flow cytometry analysis showed a decrease in the proportion of CD3E^+^ and NK1.1^+^ NKT cells 1 day after topical application of drug-loaded hydrogel (Fig. 7F and fig. S14). Immunofluorescence staining confirmed the reduced number of CD3E^+^ and CD161^+^ cells (Fig. 7, G and H). The drug-loaded hydrogel also reduced the inflammatory factors Tumor Necrosis Factor Alpha (TNFA) and IL1B as observed by immunofluorescence staining (fig. S15, A to D). μCT reconstruction showed that topical application of NK1.1 antibody hydrogel promoted new bone formation in the extraction wound (Fig. 7I). H&E staining also confirmed the same histological change in bone mass (Fig. 7I). Quantitative analysis of μCT data showed that the BMD, BV/TV, and Tb.Th were increased, and Tb.Sp was decreased in the drug-loaded hydrogel group (Fig. 7J).

## DISCUSSION

Once injured, a topical hematoma forms at the bone trauma site, releasing large amounts of inflammatory factors to induce immune cells infiltration into the injury site. Then, the regenerative cascade reaction is activated, which generates the early inflammatory microenvironment of bone injury (*19*, *20*). However, because of the diversity of immune system and the rapid remodeling in inflammatory responses at different stages, the dynamic changes of the immune microenvironment in bone injury repair are still poorly understood.

In clinical practice, bone injuries, especially open wounds caused by trauma, are often accompanied by wound infection, and infection at the injury site will lead to a stronger inflammatory response and delay bone healing. Alveolar bone defect caused by tooth extraction is one of the most common open bone injuries. The specific anatomic structure of mouse alveolar bone is able to provide a more consistent tissue for scRNA-seq compared to the soft hematoma tissue in long bone fractures. Thus, we chose tooth extraction injury as a model to explore the dynamic changes in the microenvironment during different stages of bone healing process.

In this study, we performed scRNA-seq on bone tissue before injury and 1 hour, 1 day, 2 days, and 7 days after injury to construct a dynamic cell atlas of the bone healing process. T cell was the cell type with the most obvious quantitative changes, which were recruited on 1 day and decreased from 2 days. These results suggested that the inflammatory response at injury site peaked at 1 day and then subsided gradually. Similar to our findings, Cho *et al.* (*21*, *22*) speculated that the acute inflammatory phase peaked at 1 day after bone injury and gradually subsided within 1 week by detecting the expression levels of several typical inflammatory factors. Intercellular communication analysis also showed that the interaction strength between T cells and other cells increased, indicating that the rapidly increasing T cells might play an important role in regulating bone repair at the early stage.

Different from the regeneration process, it is generally believed that infiltrating lymphocytes mainly play a role in controlling infection and clearing tumor cells (*23*). Intense inflammation following tissue injury may have evolved as a response to pathogens, but exposure to excessive inflammation can adversely affect repair processes. T lymphocytes have been observed to be one of the earliest cell types to be recruited in the early stage of bone repair and played an important role in regulating the early inflammatory response at the injury site (*24*). In *Rag*^−/−^ mice lacking T and B lymphocytes, faster healing of fracture injuries and lower expression levels of inflammatory cytokines were observed (*24*). Different subsets of T cells also have different regulatory effects on bone healing. CD8^+^ cytotoxic T cells have been shown to negatively regulate the bone repair process (*25*), whereas T_reg_ cells are believed to reduce inflammation and promote bone repair (*26*). Therefore, we performed subcluster analysis on T cell and found that NK1.1-positive NKT cell was the cell type with the highest proportion among T cells, and its proportion also reached the highest on 1d after injury.

NKT cells are known as an immune cell population with the characteristics of both NK cells and T cells. Activated NKT cells can secrete a variety of cytokines and directly or indirectly participate in the body’s immune response (*27*). NKT cells have been reported to promote B cell humoral immune responses during viral infections (*28*). Activated NKT cells can secrete large amounts of inflammatory factors and exert cytotoxic effects against malignant cells via FasL and perforin/granzyme pathway (*29*, *30*). Chimeric antigen receptor NKT cell therapy has achieved exciting clinical results in cancer treatment (*31*). The above evidence suggests the important role of NKT cells in regulating inflammatory responses. However, the role of NKT cells in bone repair has not been reported so far.

Although the natural ligands for NKT cells remain to be identified, some microorganisms have been shown to induce NKT cell activation (*32*, *33*). After bone injury, tissue damage and bacterial infection lead to cell death and inflammatory responses, which may produce a series of self- or bacterial-derived lipid antigens. Subsequently, antigen-presenting cells (APCs) capture and process lipid antigens and the processed lipid antigens are loaded onto CD1d molecules and expressed on the cell surface. NKT cells specifically recognize this lipid antigen-CD1d complex and thereby receive activation signals. However, the specific activation mechanism of NKT cells in alveolar bone injury has not yet been reported. The relevant molecular mechanisms and signaling pathways are needed to be explored in future studies.

Activated NKT cells may secrete a variety of cytokines to exert regulatory effects. In our study, treatment with NKT CM in vitro attenuated the osteogenic differentiation potential of MSCs. Depletion of NKT cells in vivo by administration of neutralizing antibodies and genetic knockout can improve bone healing. NKT cells have been reported to differentiate into NKT1, NKT2, and NKT17 cells (*34*). Considering the potential contribution of different NKT cell populations to bone repair, we compared the expression of classic type 1, type 2, and type 17 cytokines. Among NK1.1-positive cells, most cells expressed type 1 cytokines but not type 2 and type 17 cytokines. Therefore, we believe that early infiltrating NKT cells may be primarily pro-inflammatory type 1 NKT cells and will impair new bone formation. In addition, in some mouse strains, mucosa-associated invariant T (MAIT) cells may also express NK1.1 (*35*). Therefore, besides NKT cells, future studies are expected to explore the potential contribution of MAIT cells to bone repair.

The chemokine CXCL2, also known as macrophage inflammatory protein 2 alpha, affects the recruitment and activation of neutrophils by binding to and activating its specific receptors CXCR1 and CXCR2, acting as an important inflammatory mediator. The expression of CXCL2 was negatively correlated with BMD (*17*). Neutralization of CXCL2 prevented bone destruction in lipopolysaccharide-challenged mice (*36*), while supplement of CXCL2 in culture medium led to cellular senescence and impaired differentiation of MSCs (*37*). In our study, differential gene analysis showed that the expression of *Cxcl2* was significantly up-regulated at 1 day. Administration of CXCL2-neutralizing antibody or *Cxcl2* knockout could partially rescue the inhibition of MSC osteogenic differentiation by NKT CM, which demonstrated that NKT cell–derived CXCL2 could influence bone repair by inhibiting the osteogenic differentiation of MSCs.

Notably, administration of CXCL2-neutralizing antibodies in *Traj18*^−/−^ mice also improved the bone repair process. This result suggested that NKT cells were not the only source of CXCL2 in the bone injury microenvironment. Besides *Cxcl2*, the expression of several other cytokines also increased in NKT cells at 1 day. Neutralizing these cytokines (IFNG, TNFSF9, and IL1B) also ameliorated the osteogenic differentiation to some extent, suggesting that NKT cells might affect osteogenic differentiation and bone repair through multiple pathways.

Our results suggested that a significant inflammatory response appears in the first day after bone injury, but most drug delivery material carriers are designed to prolong the drug delivery time as much as possible to match the entire repair cycle of the tissue. Because of the rapid changes in immune cell populations during the acute inflammatory phase of bone injury, long-term sustained-release drug-loading system may not be able to accurately match the therapeutic targets and result in waste of loaded drugs. Moreover, although depletion of NKT cells through systemic administration or gene editing can accelerate bone healing, these strategies lack clinical practicability. Therefore, we hope to achieve accelerated bone healing by topical drug delivery. Gelatin is a hydrolyzed product derived from collagen, which exhibits ideal biocompatibility and low antigenicity (*38*), and is widely used in tissue engineering. Its derivative, GelMA hydrogel, is an ideal platform for tooth extraction wound repair and drug delivery for its injectable and photocrosslinking properties, which can adapt to various shapes of tissue defects. Drug release periods in GelMA hydrogels have been reported ranging from a few days to a month (*39*, *40*). Here, we introduced micron-sized pores into the hydrogel to increase the contact area with body fluid environment. The introduction of microporous structure accelerated the drug release rate of GelMA hydrogel, which better matched the rapid inflammation and NKT cell infiltration after bone injury. The drug-loaded pGelMA hydrogel effectively reduced topical infiltrating NKT cells and accelerated the healing process of alveolar bone, which illustrated the feasibility of topical drug delivery targeting early infiltered NKT cells.

In this study, we used mouse model to investigate bone injury, which usually heals within 4 weeks. This process in large animals or human beings is much longer, thus the specific duration of acute inflammation may vary under different conditions. In addition, given the significant differences between NKT cell populations in human and in mice, more studies based on clinical samples are needed to further validate the role of NKT cells in bone injury healing.

In summary, we discover a rapid infiltration of NKT cells after alveolar bone injury in mice, and it interacts with MSCs to attenuate bone regeneration. Our data suggest that targeting the regulation of early inflammatory stage represented by NKT cell infiltration can accelerate bone healing and may serve as a potential therapeutic strategy.

## MATERIALS AND METHODS

### Animal models

WT C57BL/6J mice and *Traj18*^−/−^ and *Cxcl2*^−/−^ mouse were purchased from Shanghai Nanfang Model Organisms Center. All mice were kept in a specific pathogen–free environment with a temperature of 20° to 25°C, a relative humidity of 50 to 70%, and 12 hours of light per day. The mouse experiments were in strict compliance with the procedures defined by the Subcommittee on Research and Animal Care of Sichuan University.

NK1.1 depletion antibody (Bio X Cell) and CXCL2-neutralizing antibody (Invitrogen) were intraperitoneally injected on days −3, 3, and 10 according to the manufacturer’s instructions. Control mice received isotype antibody.

### Single-cell RNA-seq

We performed scRNA-seq of the mouse alveolar bone before tooth extraction (Ctrl), after 1 hour, 1 day, 2 days, or 7 days after tooth extraction. Four 8-week-old male C57BL/6J mice were pooled to prepare single-cell suspensions according to our previously published study (*14*). Briefly, the mandibles were isolated under a stereomicroscope, and muscle and gingival tissue were carefully removed. The molars were gently loosed by 26-gauge syringe needle and slowly removed using forceps. The alveolar bones were obtained by scissoring along the upper side of the incisor root, and the condyles and coracoid processes were cut off. The alveolar bones were minced into less than 1-mm^3^ fragments and digested in α-minimum essential medium (αMEM) medium containing collagenase II (1 mg/ml), dispase (1 mg/ml), and deoxyribonuclease I (DNase I) (10 U/ml) at 37°C for 45 min and then filtered through a 100-μm cell mesh. The filtered cells were treated with erythrocyte lysate for 1 min followed by twice wash with phosphate-buffered saline (PBS). The cell suspensions were allowed to settle for 2 min before discarding the bottom 100 μl of the suspension. Mg^2+^- and Ca^2+^-free PBS was used to adjust the cell concentration to 800 to 1500 cells/μl. About 20,000 cells were loaded on the instrument to capture approximately 10,000 target cells following the manufacturer’s instructions, and cDNA library was constructed using Chromium Single Cell v3.0 reagents. The cDNA library was stored in a −80°C refrigerator and sequenced on the Illumina NovaSeq system.

### scRNA-seq data processing and analysis

The R package Seurat was used for quality control, dimensionality reduction, and clustering of scRNA-seq data (*41*, *42*). Cells with gene number less than 6000 and greater than 200 and mitochondrial gene number less than 25% were considered as high-quality cells for inclusion in the next analysis. After normalization, the FindVariableFeatures() function was used to select high-variable genes. Principal components analysis and Uniform Manifold Approximation and Projection were conducted for dimension reduction and visualization. Identification of alveolar bone cell populations was performed following our previously published study (*14*). For differential gene analysis, we used the FindAllMarker() function in the Seurat package for calculation. The CellChat package was used to analyze cell communication in different subpopulations (*43*). The Slingshot package was used for pseudo-time analysis (*44*). GO enrichment analysis was performed on an online website metascape (*45*). For cellular proportion analysis, we utilized the scDC package (*46*) to infer the cell-type proportion via bias-corrected and accelerated bootstrap confidence intervals.

### μCT analysis

μCT analysis was performed as previously described (*47*). The Nemo μCT (NMC-100) equipment was used for scanning, and the three-dimensional image was reversely reconstructed by the Feldkamp-Davis-Kress (FDK) method on the Avatar software (version 1.6.2, PINGSENG Healthcare Incorporation). Cylindrical area in the tooth extraction socket and distal femur segment area were selected as the area of interest, and then the BMD, BV/TV, Tb.Th, and Tb.Sp values were calculated.

### Flow cytometry analysis

The alveolar socket and nearby bone tissue were collected at different time points before and after tooth extraction. After the tissue was minced, it was digested in αMEM medium containing collagenase II (1 mg/ml), dispase (1 mg/ml), and DNase I (10 U/ml), and incubated at 37°C for 45 min. Samples were filtered using a 100-μm cell mesh. The centrifuged cells were treated with erythrocyte lysate for 1 min and then centrifuged again. Cells were stained with fluorescently labeled antibodies (anti-CD45, anti-CD3E, and anti-NK1.1) (BD Pharmingen) and mouse CD1d tetramer (MBL Biotech) in 100 μl of PBS for 30 min on ice. Labeled cells were then washed and detected on a flow cytometer (Invitrogen).

### Histological staining

The scanned tissue samples were decalcified in 10% EDTA solution for 4 weeks at room temperature. The samples were then dehydrated and embedded. Tissue sections were prepared parallel to dentition with a paraffin microtome (Leica).

For H&E staining, sections (5 μm thick) were cut, deparaffinized, and rehydrated in graded ethanol solutions. Sections were stained with hematoxylin and eosin according to the instruction and then dehydrated and mounted with neutral resin. The healing process of the extraction socket was observed by optical microscopy (Olympus). For immunofluorescence staining, sections (5 μm thick) were cut, deparaffinized, and rehydrated in graded ethanol solutions. After antigen retrieval in sodium citrate buffer, sections were blocked with 3% bovine serum albumin solution and incubated for 30 min. Primary antibodies were added to incubate overnight at 4°C and then incubated with secondary antibodies for 1 hour. Cell nuclei were stained with 4′,6-diamidino-2-phenylindole and mounted using glycerol. Observation and photography were performed with a confocal laser scanning microscope (Olympus), and the proportion of double-positive cells was analyzed using ImageJ statistical software.

### Primary cell isolation and culture

Bone MSCs were extracted as previously described (*48*). Briefly, bilateral femurs and tibias from 6 -to 8-week-old WT mice were dissected. The bone marrow cavity was flushed, and the cells were cultured in αMEM medium containing 10% fetal bovine serum (FBS). After 24 hours, the medium was replaced to remove nonadherent cells. The medium was then changed every 2 days.

For NKT cells, spleens form 6- to 8-week-old WT mice were crushed on a 100-μm cell mesh and rinsed with PBS. To activate NKT cells, mice received an intraperitoneal injection of 1 μg of α-galcer (MedChemExpress Incorporation) in 200 μl of PBS 24 hours before spleen cell collection (*49*). We performed magnetic bead sorting of NKT cells following the instructions of the NKT Cell Isolation Kit (Miltenyi). Briefly, non-NKT cells were first labeled with biotin-conjugated cocktail antibody and incubated on ice for 10 min and then incubated with anti-biotin magnetic beads for another 10 min. LD columns were used for negative selection. The obtained cells were washed with PBS and incubated with NK1.1-APC antibody for 10 min. After adding anti-APC magnetic beads, NKT cells were obtained by positive selection using LS column and cultured with 10% FBS Dulbecco’s modified Eagle’s medium (DMEM) medium. For cell migration experiments, 1% FBS DMEM was used to eliminate the effect of serum.

### CCK8

First, MSCs were seeded into 96-well plates at a density of 5 × 10^3^ cells per well, and the complete medium with or without NKT CM was replaced every 2 days. Detection was performed on 1, 3, and 5 days. Culture medium containing 10% CCK-8 (Dojindo Laboratories) solution was replaced in each well. After incubating at 37°C for 1 hour, the absorbance at 450 nm of the supernatant of each group was detected using a microplate reader (Thermo Fisher Scientific), and the cell viability of each group was calculated.

### Colony formation test

MSCs were seeded into 6-well plates at a density of 500 cells/well, and the complete medium with or without NKT CM was replaced every two days. On day 14, cells were fixed with 4% paraformaldehyde solution and stained with 0.1% crystal violet solution. Observe and take pictures under an optical microscope (Olympus), and count the number of cell colonies in each well (>30 cells).

### β-Gal staining

MSCs were first seeded into six-well plates, then cultured with normal complete medium and NKT CM for 3 days, and then stained according to the instructions of the β-galactosidase detection kit (Beyotime). Cells were washed with PBS and fixed with 4% paraformaldehyde for 5 min. Senescence-associated β-galactosidase (SA–β-gal) staining solution was added to stain senescent cells and incubated at 37°C for 12 hours. Three fields of view were randomly selected for observation under an optical microscope (Olympus).

### Cell migration assay

For the scratch assay, MSC cells were seeded into six-well plates at a density of 2 × 10^6^ per well and cultured until the cell confluency reached 100%. Then, the complete medium was replaced with medium containing 1% FBS and starved for 24 hours. A 20- to 200-μl pipette tip were used to make a scratch on each well along a straight ruler. A 1% FBS medium and NKT cell conditioned medium were added for culture. After 0, 24, and 72 hours of culture, photographs were taken with an optical microscope (Olympus), and the percentage of scratch healing was calculated using ImageJ software.

For transwell assay, transwell chambers (Corning) with pore size of 8 μm were placed on a 24-well plate, and 1 × 10^4^ cells in 200 μl of serum-free medium with or without NKT CM were added to each well in the upper chamber. A total of 600 μl of DMEM medium with 10% FBS was added in the lower chamber and cocultured for 24 hours. After washing with PBS, the cells were fixed with 4% paraformaldehyde solution for 20 min, and the unmigrated cells in the upper chamber were wiped off with a cotton swab and washed with PBS again. A total of 1 ml of 0.1% crystal violet solution was added in each well for staining. Observation and photographing were carried out under an optical microscope (Olympus). The number of cells migrating to the lower chamber were counted by ImageJ software.

### Osteogenic differentiation induction

For osteogenic differentiation induction, MSCs were seeded into a 24-well plate with a density of 5 × 10^4^ cells per well. The αMEM complete medium was supplemented with ascorbic acid (concentration of 50 μg/ml), β-glycerophosphate (concentration of 5 mM), and dexamethasone (concentration of 100 nM). Neutralizing antibodies of CXCL2 (Invitrogen), IFNG, TNFSF9, and IL1B (BioLegend) were added to the culture medium at concentrations recommended by the manufacturer.

ALP staining was performed according to the instructions of the ALP staining kit (Beyotime). After 7 days of osteogenic induction, the cells were fixed with 4% paraformaldehyde and washed with PBS. Then, ALP staining solution was added and stained for 30 min. The ALP staining of cells in each group was scanned with a scanner (Epson). According to the instructions of the ALP activity detection kit (Beyotime), lysate buffer was added to each group for 10 min. Then, the supernatant, chromogenic substrate, and *p*-nitrophenol were co-incubated at 37°C for 20 min. Absorbance at 405 nm was detected by a microplate reader (Thermo Fisher Scientific). At the same time, the total protein content of each group was measured using the Bicinchoninic Acid Protein Assay Kit (Beyotime), and the normalized ALP relative activity was calculated.

To perform ARS staining, cells were fixed with 4% paraformaldehyde after 14 days osteogenic induction. 1% ARS staining solution was co-incubated with cells for 10 min. After washing with PBS, the formation of mineralized nodules in the cells of each group was observed under an optical microscope (Olympus) and photographed. For semi-quantitative analysis, 1 ml of 10% cetylpyridinium chloride (Sigma) solution was added to each well to dissolve alizarin red, and the supernatant after shaking and incubating for 30 min was detected by a microplate reader (Thermo Fisher) at a wavelength of 562 nm.

### Real-time quantitative PCR

After the fifth day of osteogenic induction, TRIzol was used to extract the total RNA, and the NanoDrop 2000 instrument (Thermo Fisher Scientific) was used to detect the purity and concentration of RNA samples. Genomic DNA in the total RNA sample was removed, and cDNA was synthesized by reverse transcription and stored at −20°C. Using cDNA as a template, reverse transcription quantitative polymerase chain reaction experiments were performed to detect the mRNA expression levels of *Runx2*, *Osx*, *Bglap*, and *Col1a1*.

### Enzyme-linked immunosorbent assay

The levels of CXCL2 in the alveolar bone at different time points were detected according to the instructions in the ELISA Detection Kit (Cusabio). Alveolar bone tissue was minced and diluted with 1 ml of buffer, then frozen, and thawed three times at −80°C. After centrifugation at 800 rpm for 5 min, 100 μl of the supernatant was added to the detection well plate, and after incubation at 37°C for 2 hours, the absorbance was detected with a microplate reader (Thermo Fisher Scientific), and the level of CXCL2 was calculated.

### Preparation of pGelMA hydrogels

Methacrylated gelatin was prepared as previously described (*50*, *51*). Briefly, 10 g of gelatin powder (Sigma-Aldrich) was dissolved in 100 ml of PBS at 50°C. After the gelatin powder was completely dissolved, 5 ml of methacrylic anhydride solution (Sigma-Aldrich) was slowly added to the gelatin solution with constant stirring and reacted for 2 hours. The product was dialyzed for 3 days and freeze-dried. To prepare the pGelMA hydrogel, 0.5 g of GelMA and 25 mg of photoinitiator lithium phenyl-2,4,6-trimethylbenzoylphosphinate were added into a light-proof centrifuge tube and dissolved in 5 ml of PBS solution. For drug loading, NK1.1 depletion antibody was mixed into the GelMA solution. A 0.16 g of polyethylene oxide (PEO) powder was added into 10 ml of PBS to obtain 1.6% PEO solution. The GelMA solution and PEO solution were mixed evenly at a volume ratio of 1:1 to obtain two-phase emulsion. The mixed solution was cured with 405-nm light after being injected around the wound. PEO droplets in bulk gel will gradually dissolve in the body fluid environment. For SEM scanning, the hydrogels were freeze-dried for 3 days, and then the microstructure was characterized.

### Statistical analyses

All data were expressed as mean ± SD. Unpaired two-tailed Student’s *t* test was used for comparison between two groups. Data between multiple groups were compared with one-way analysis of variance (ANOVA) followed by the Tukey’s post hoc test. *P* < 0.05 was considered as statistically significant. GraphPad Prism 9 software was used for data analysis and figure generation.
