# Supplementary material for: Early infiltrating NKT lymphocytes attenuate bone regeneration through secretion of CXCL2
Source: Sci Adv. 2024 May 17;10(20):eadl6343. doi: 10.1126/sciadv.adl6343 (PMC11100573; doi:10.1126/sciadv.adl6343)
Supplement: Supplementary file 1 — Figs. S1 to S15 [file sciadv.adl6343_sm.pdf]

Science Advances
AAAS

Supplementary Materials for

Early infiltrating NKT lymphocytes attenuate bone regeneration through secretion of CXCL2

Weimin Lin *et al.*

Corresponding author: Malcolm Xing, malcolm.xing@umanitoba.ca;
Chenchen Zhou, chenchenzhou5510@scu.edu.cn; Quan Yuan, yuanquan@scu.edu.cn

Sci. Adv. **10**, eadl6343 (2024)
DOI: 10.1126/sciadv.adl6343

This PDF file includes:

Figs. S1 to S15

Supplementary figure 1

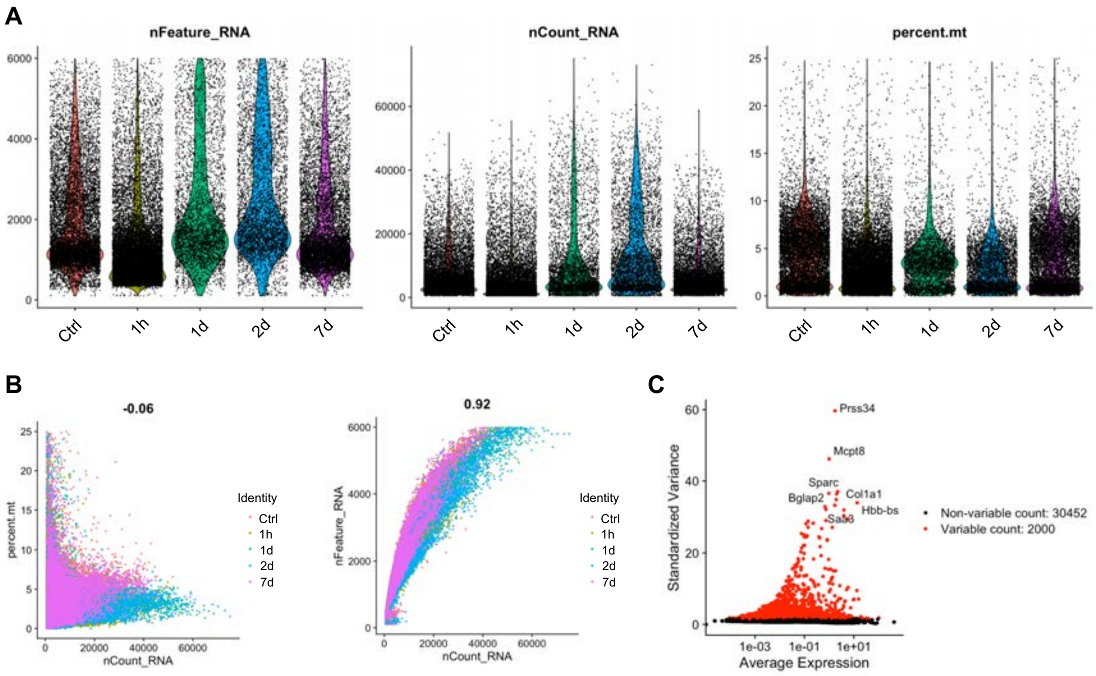

Supplementary Figure 1. Quality control of the alveolar bone marrow scRNA-seq data. (**A**) The violin plots showed the distribution of feature_RNA, count_RNA and mitochondrial RNA proportions. (**B**) FeatureScatter plot revealed the relationship between mitochondrial RNA percent and count_RNA, as well as Feature_RNA and count_RNA. (**C**) The variable feature plot showed the expression levels of 32452 genes in all cells. The 2000 genes with the most variable value were selected for subsequent analysis, and the top 5 genes were marked.

Supplementary figure 2

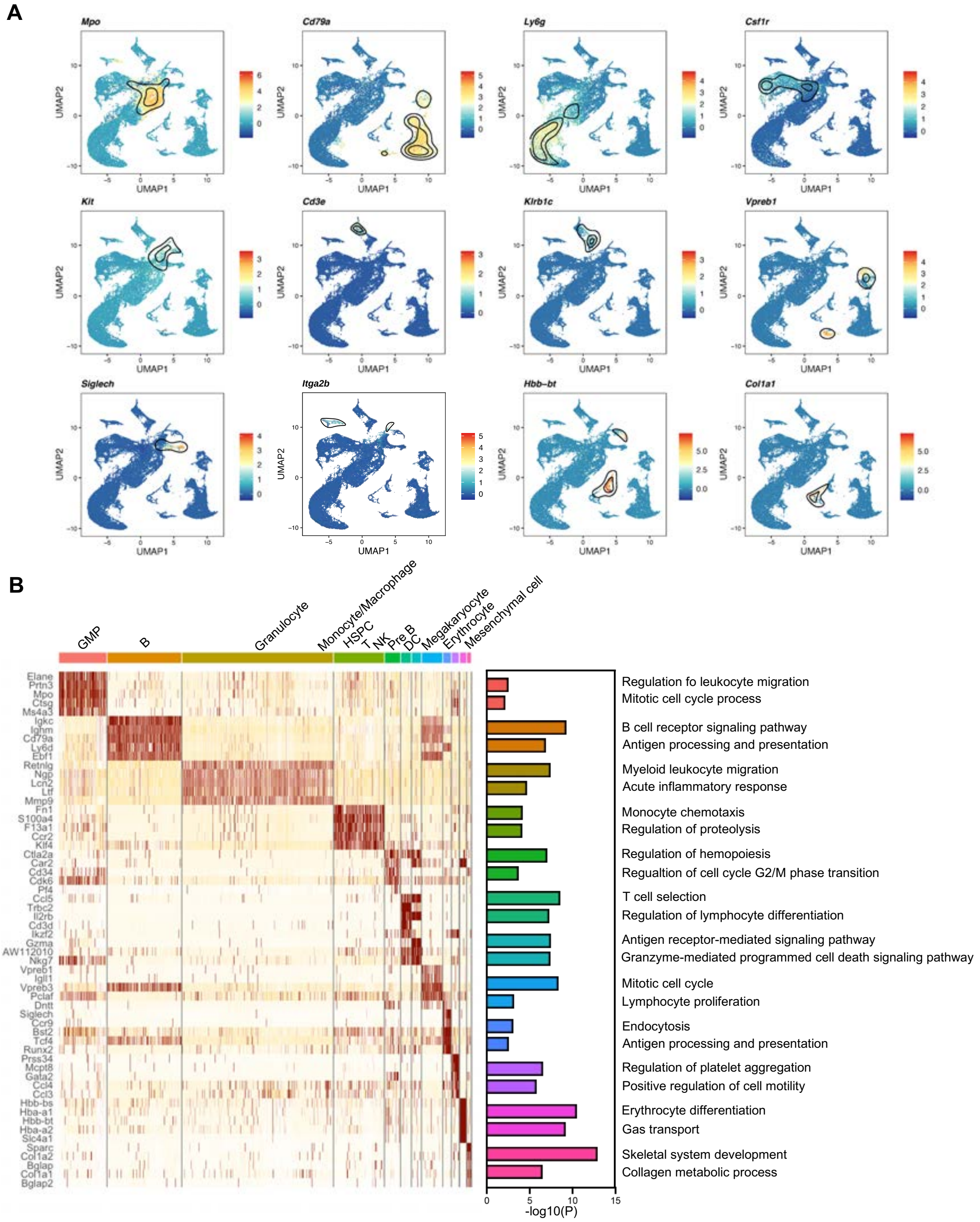

Supplementary Figure 2. Cell-type specific gene expression. (**A**) Feature plot shows the expression of marker genes in different cell populations. (**B**) Heatmap of 12 cell populations. After dividing the cells into 12 populations, top 5 genes with the highest expression in each population were identified. The representative GO enrichment analysis results were showed in bar plot.

Supplementary figure 3

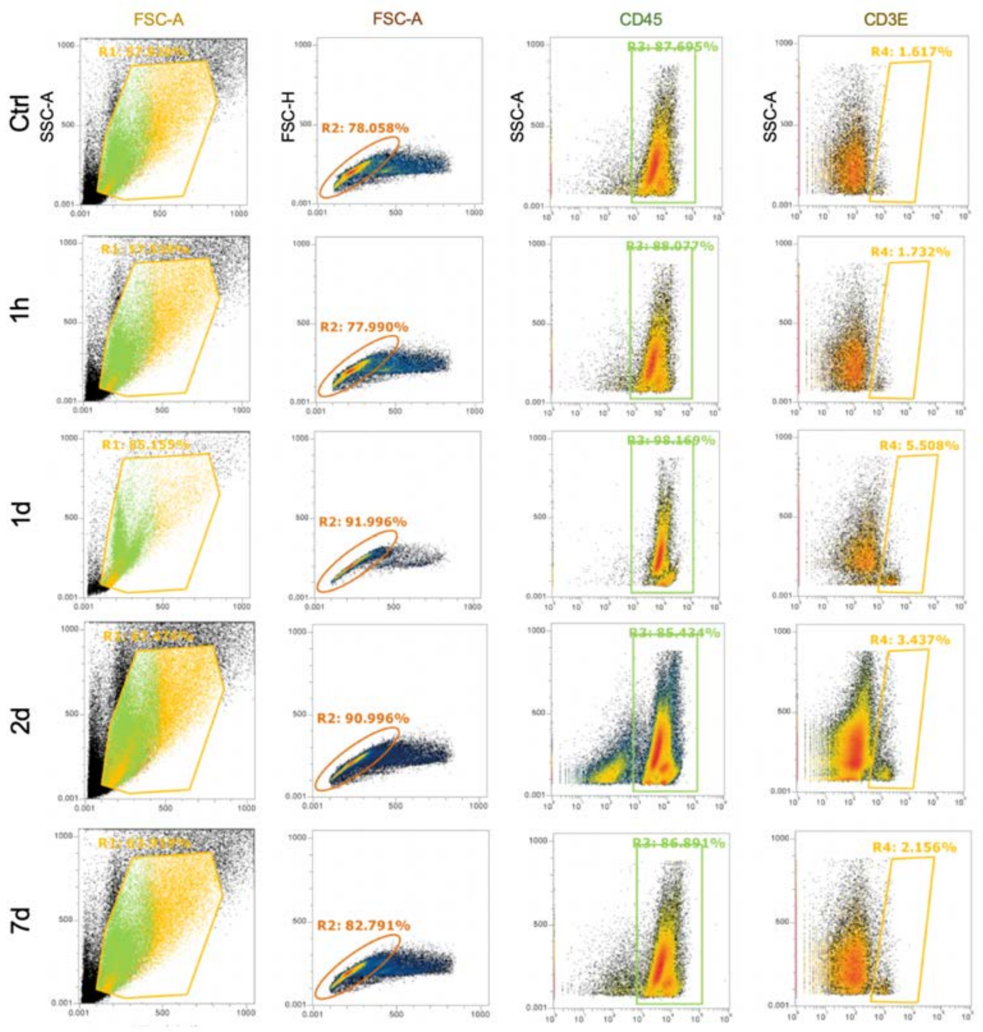

Supplementary Figure 3. Flow cytometry analysis and gating strategy on CD3E⁺ T cells at different time points

Supplementary figure 4

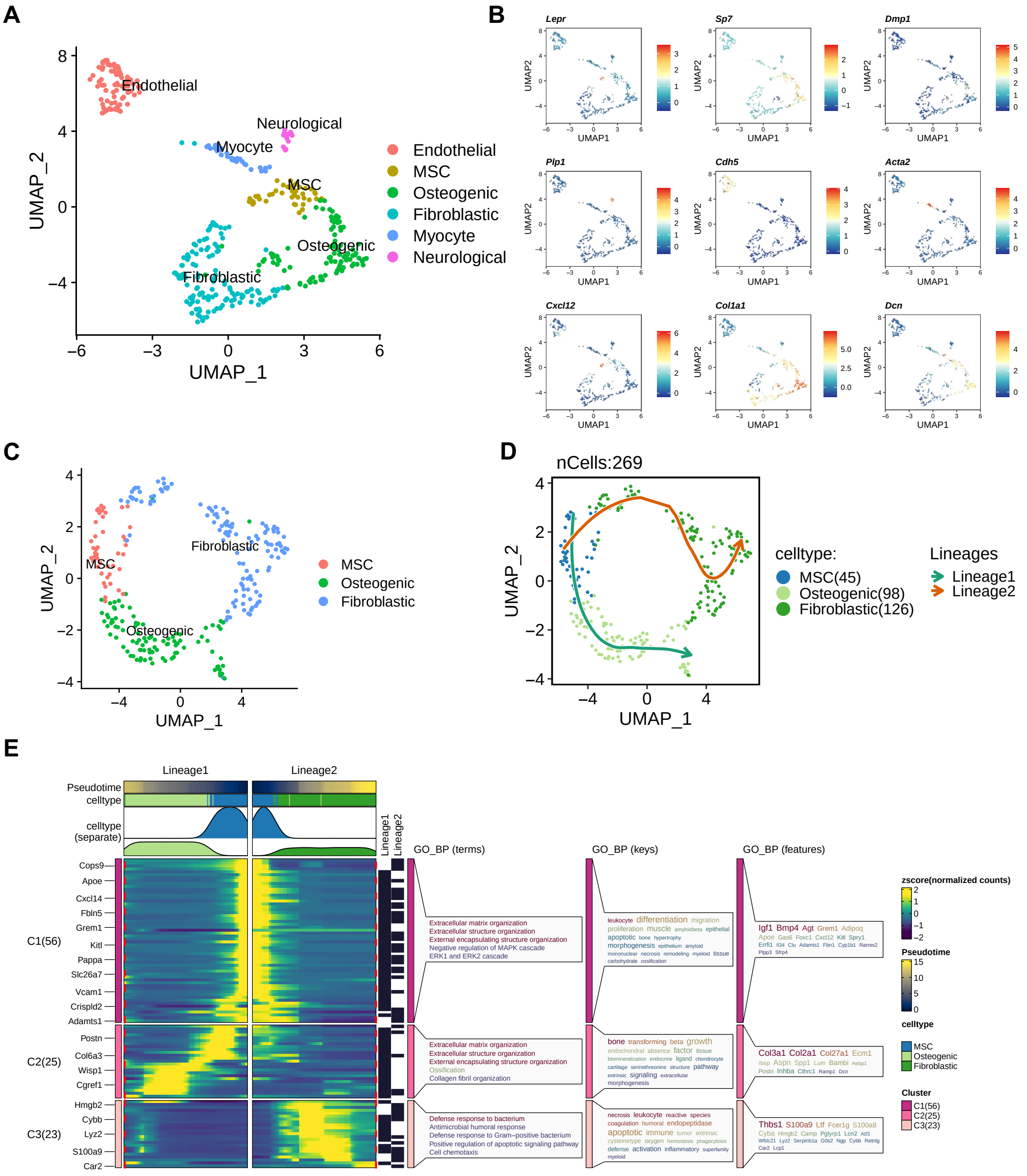

Supplementary Figure 4. Analysis of mesenchymal cell subpopulations and their differentiation trajectories. (A) Mesenchymal cells include 6 subpopulations. (B) Classic marker gene expression of different mesenchymal cell populations. (C) UMAP plot of MSC, osteogenic cells and fibroblastic cells. (D) MSC differentiation trajectory inference. (E) Clustered heatmap and enrichment of differential genes at two MSC trajectory branch points.

Supplementary figure 5

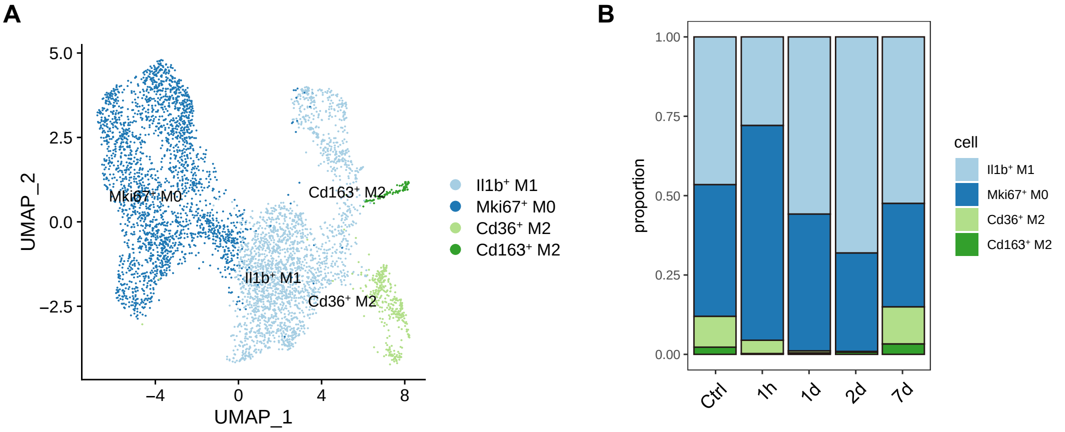

Supplementary Figure 5. Analysis of monocyte/macrophage subclusters. (A) Monocyte/macrophages include 4 subclusters. (B) Changes in the proportion of monocyte/macrophage subclusters at different time points.

Supplementary figure 6

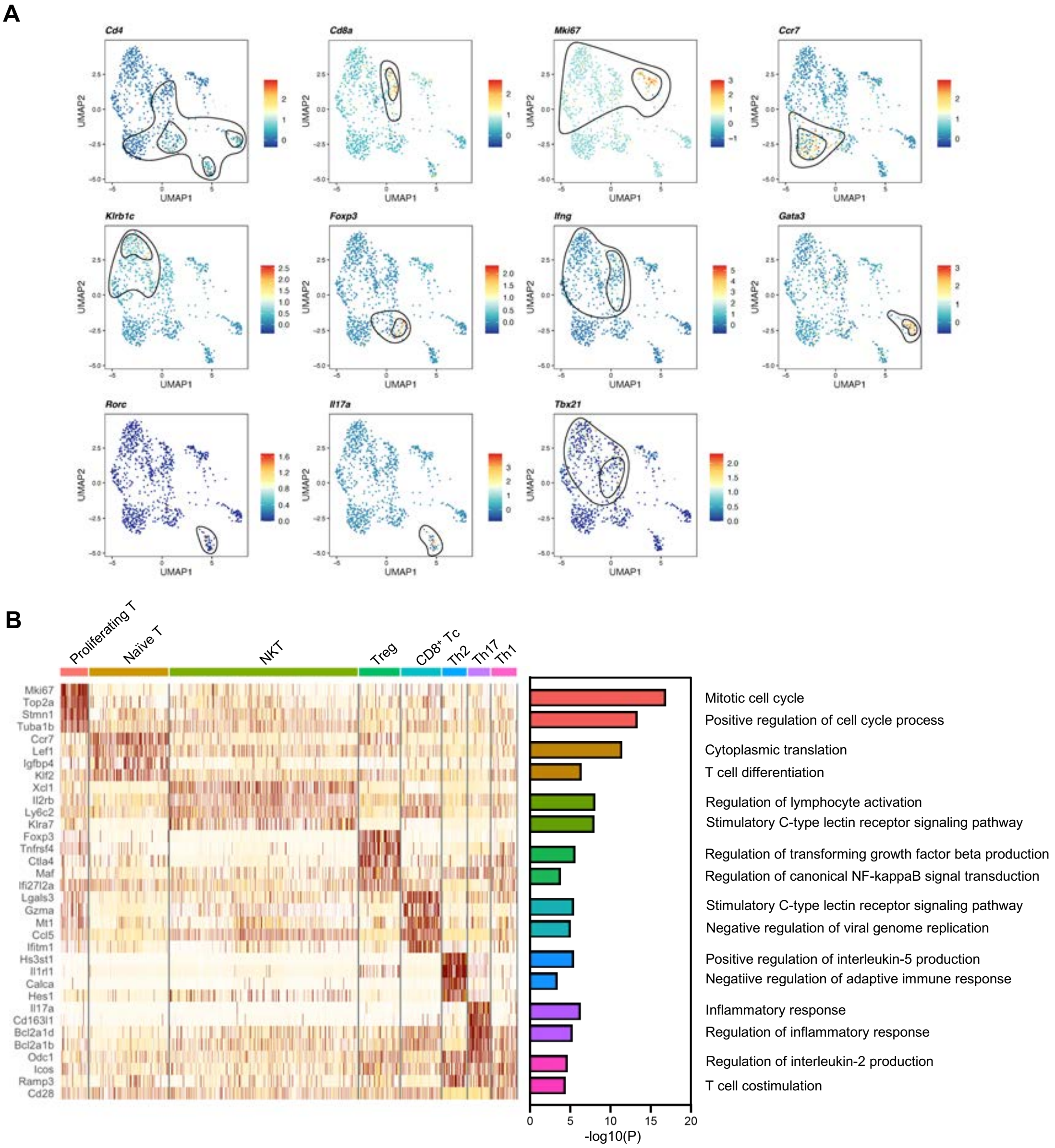

Supplementary Figure 6. Cell-type specific gene expression. (**A**) Feature plot shows the expression of marker genes in different T cell subclusters. (**B**) Heatmap of the top 5 gene expression and GO enrichment analysis of the top 50 genes in T cell subclusters.

Supplementary figure 7

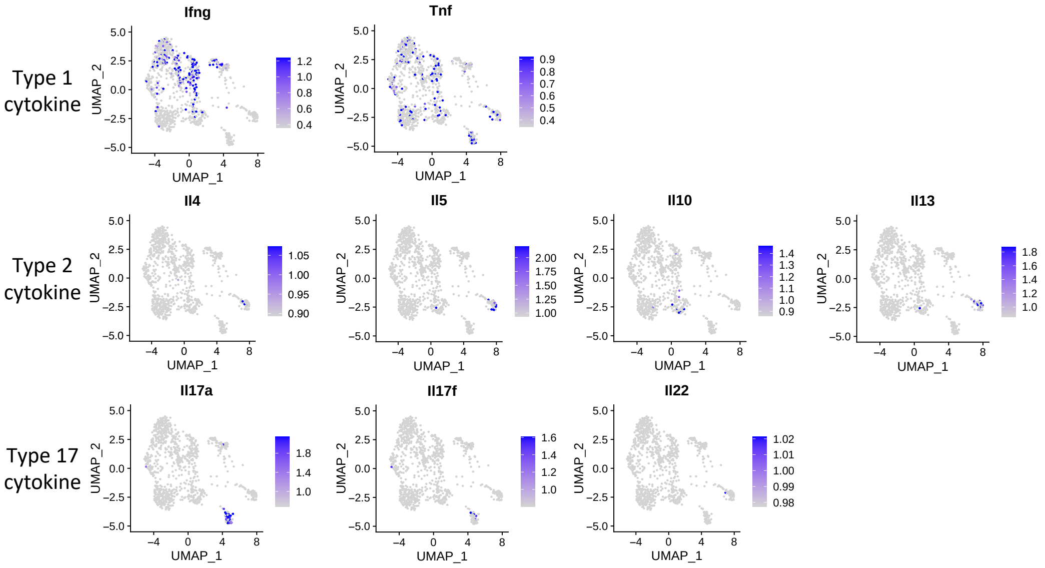

Supplementary Figure 7. Expression of type 1, type 2 and type 17 cytokines in T cells.

Supplementary figure 8

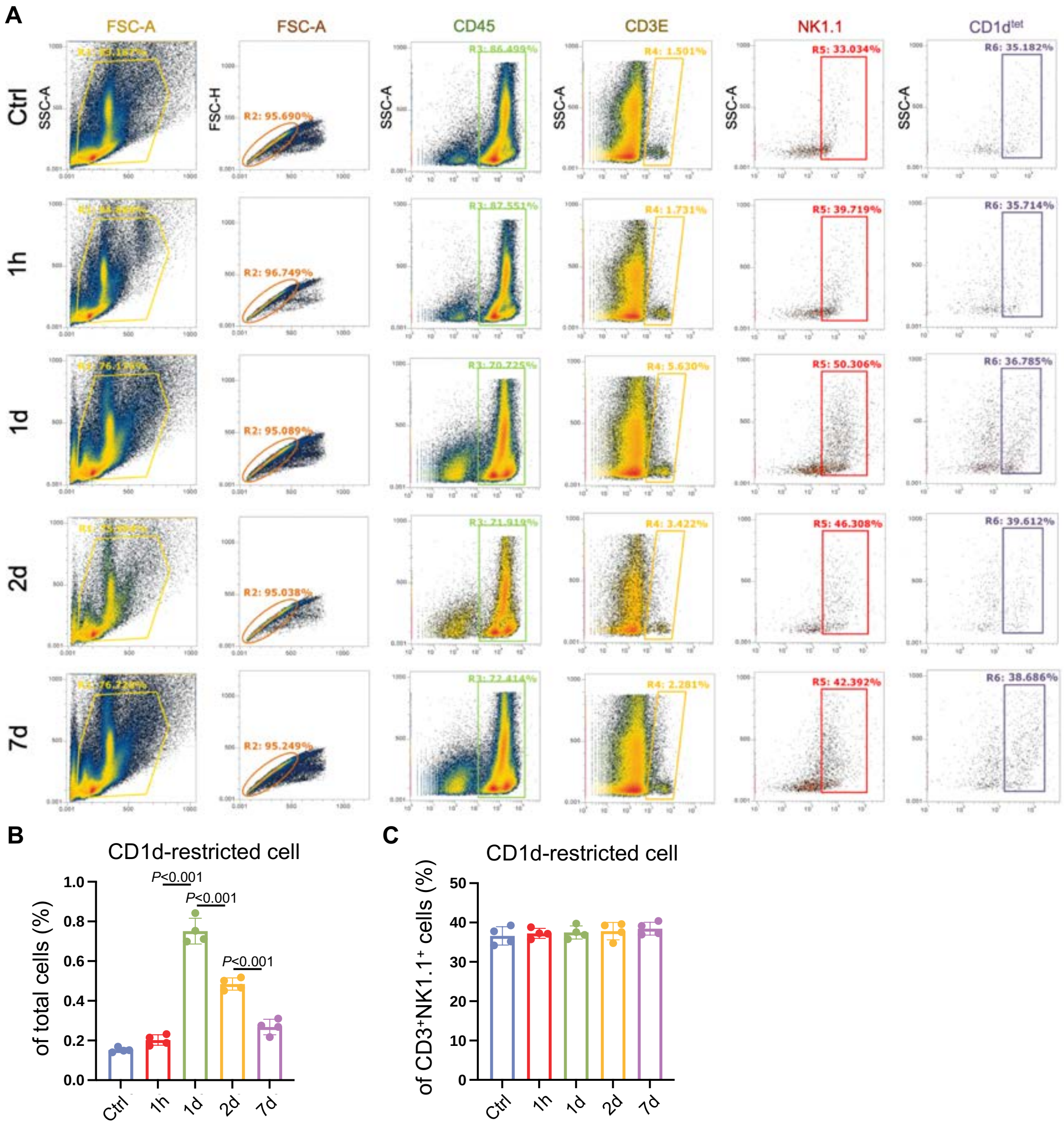

Supplementary Figure 8. Flow cytometry analysis and gating strategy on CD45⁺ CD3E⁺ NK1.1⁺ NKT cells and CD1d-restricted cells at different time points. (A) Gating strategy on CD45⁺ CD3E⁺ NK1.1⁺ and CD1d-restricted cells. (B) Statistical analysis of CD1d-restricted cell proportion of total cells. (C) Statistical analysis of CD1d-restricted cell proportion of CD3E⁺ NK1.1⁺ cells.

Supplementary Figure 9

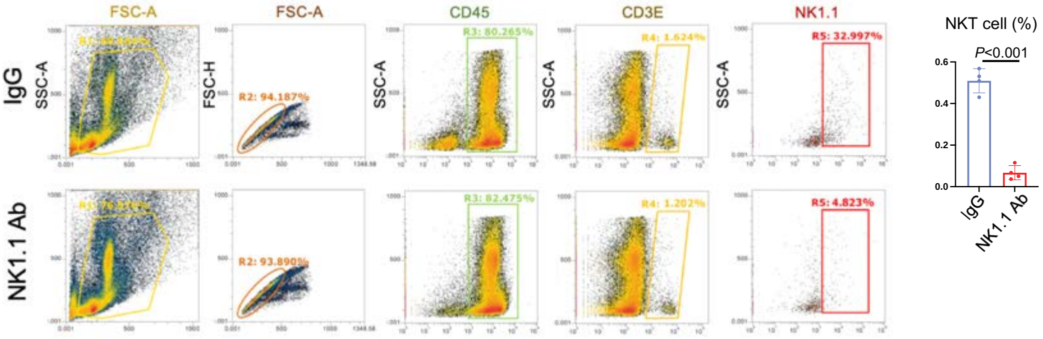

Supplementary Figure 9. Flow cytometry analysis of CD3E^{+} NK1.1^{+} NKT cells in alveolar bone marrow after NK1.1 depletion antibody injection or IgG control.

Supplementary Figure 10

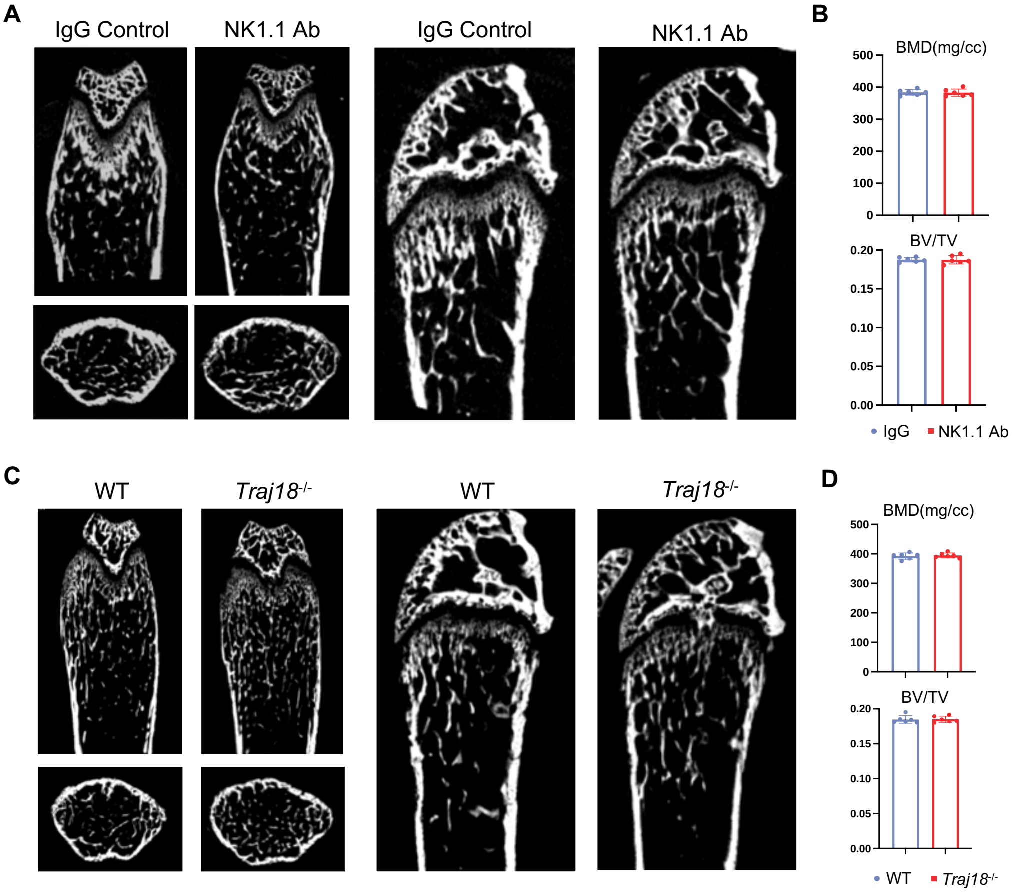

Supplementary Figure 10. The bone mass of NK1.1 depletion antibody receiving mice and *Traj18*$^{-/-}$ mice. (**A**) μCT analysis of the femur bone mass in NK1.1 depletion antibody receiving mice and IgG control. (**B**) Quantitative analysis of BMD and BV/TV. (**C**) μCT analysis of the femur bone mass in *Traj18*$^{-/-}$ mice and WT control. (**D**) Quantitative analysis of BMD and BV/TV.

Supplementary figure 11

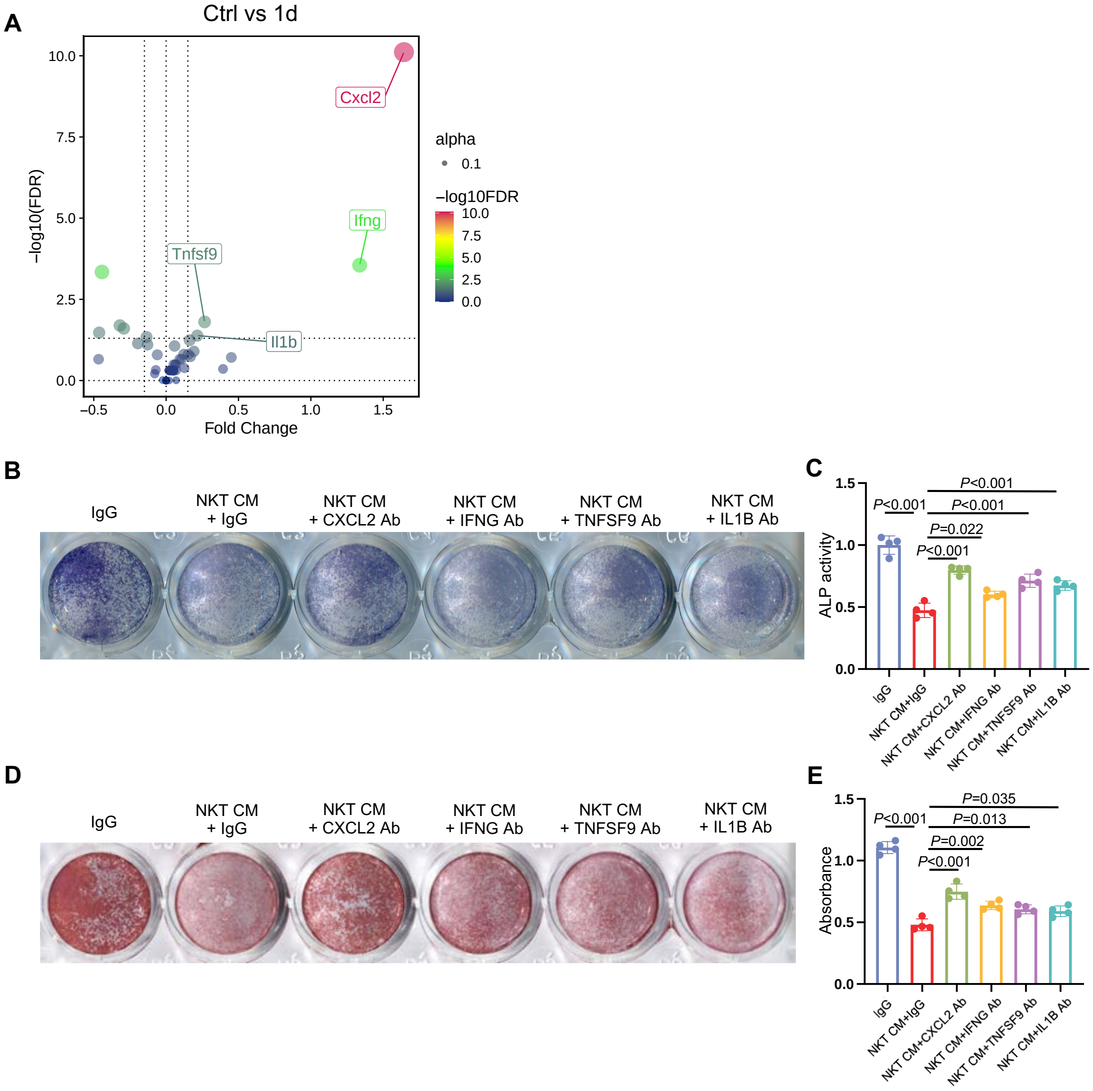

Supplementary Figure 11. Upregulated cytokines in NKT cells and their effects on osteogenic differentiation. (A) Volcano plot showed differentially expressed cytokines in NKT cells. (B) ALP staining results after treatment with different cytokine neutralizing antibodies. (C) Quantitative analysis of ALP activity. (D) ARS staining results after treatment with different cytokine neutralizing antibodies. (E) Semi-quantitative analysis of ARS staining.

Supplementary Figure 12

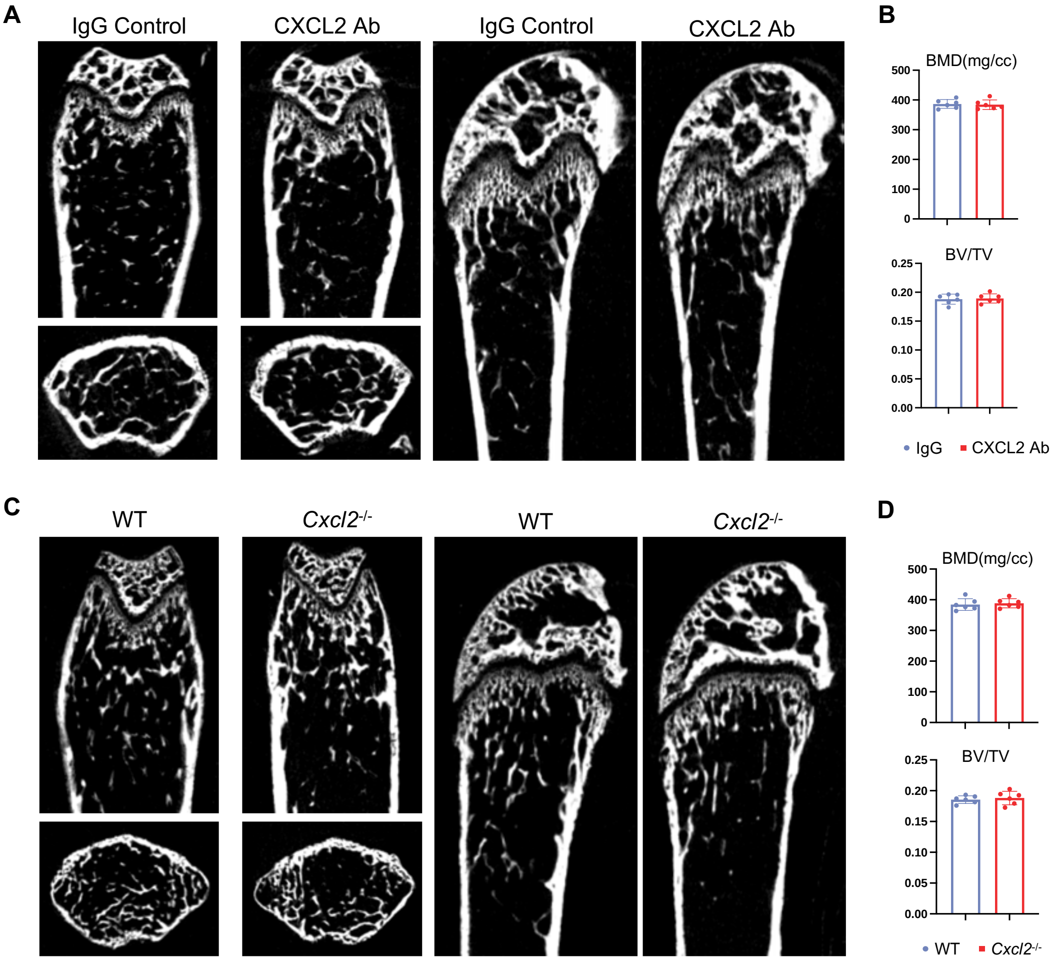

Supplementary Figure 12. The bone mass of CXCL2 neutralization antibody receiving mice and *Cxcl2*$^{-/-}$ mice. (**A**) μCT analysis of the femur bone mass in CXCL2 neutralization antibody receiving mice and IgG control. (**B**) Quantitative analysis of BMD and BV/TV. (**C**) μCT analysis of the femur bone mass in *Cxcl2*$^{-/-}$ mice and WT control. (**D**) Quantitative analysis of BMD and BV/TV.

Supplementary Figure 13

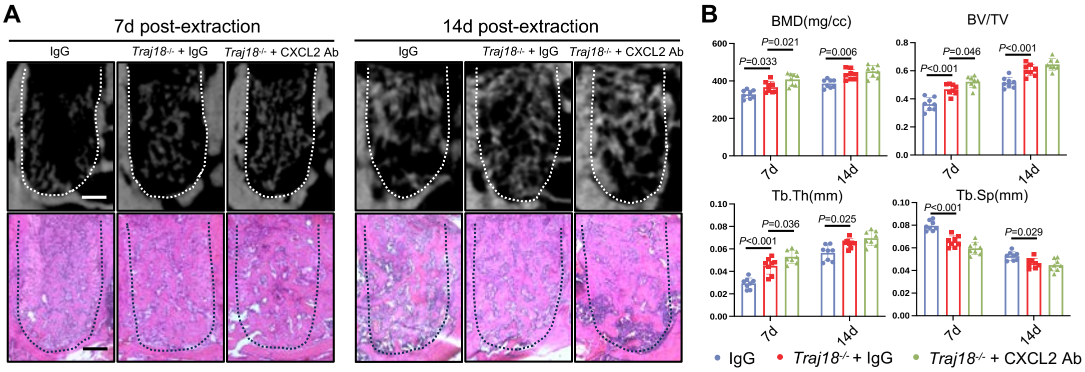

Supplementary Figure 13. Alveolar bone injury repair in *Traj18*$^{-/-}$ mice treated with CXCL2 neutralizing antibody. (A) μCT analysis and H&E staining of the alveolar bone injury area at 7 and 14 days after CXCL2 neutralizing antibody treatment in *Traj18*$^{-/-}$ mice Mouse. Scale bar = 150μm. (B) μCT quantitative analysis of regenerated bone volume at the alveolar bone injury site after CXCL2 neutralizing antibody treatment in *Traj18*$^{-/-}$ mice.

Supplementary Figure 14

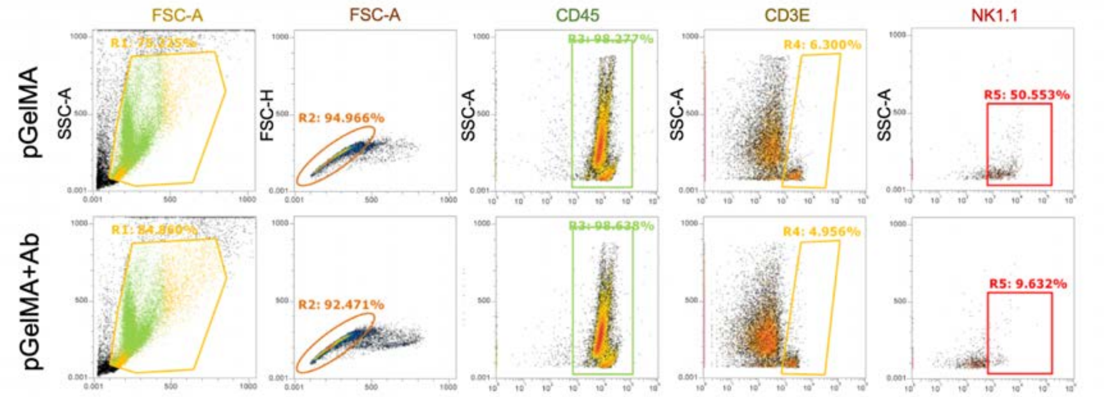

Supplementary Figure 14. Flow cytometry analysis of $CD3E^{+}$ $NK1.1^{+}$ NKT cells with or without topical application of drug-loaded hydrogel.

Supplementary Figure 15

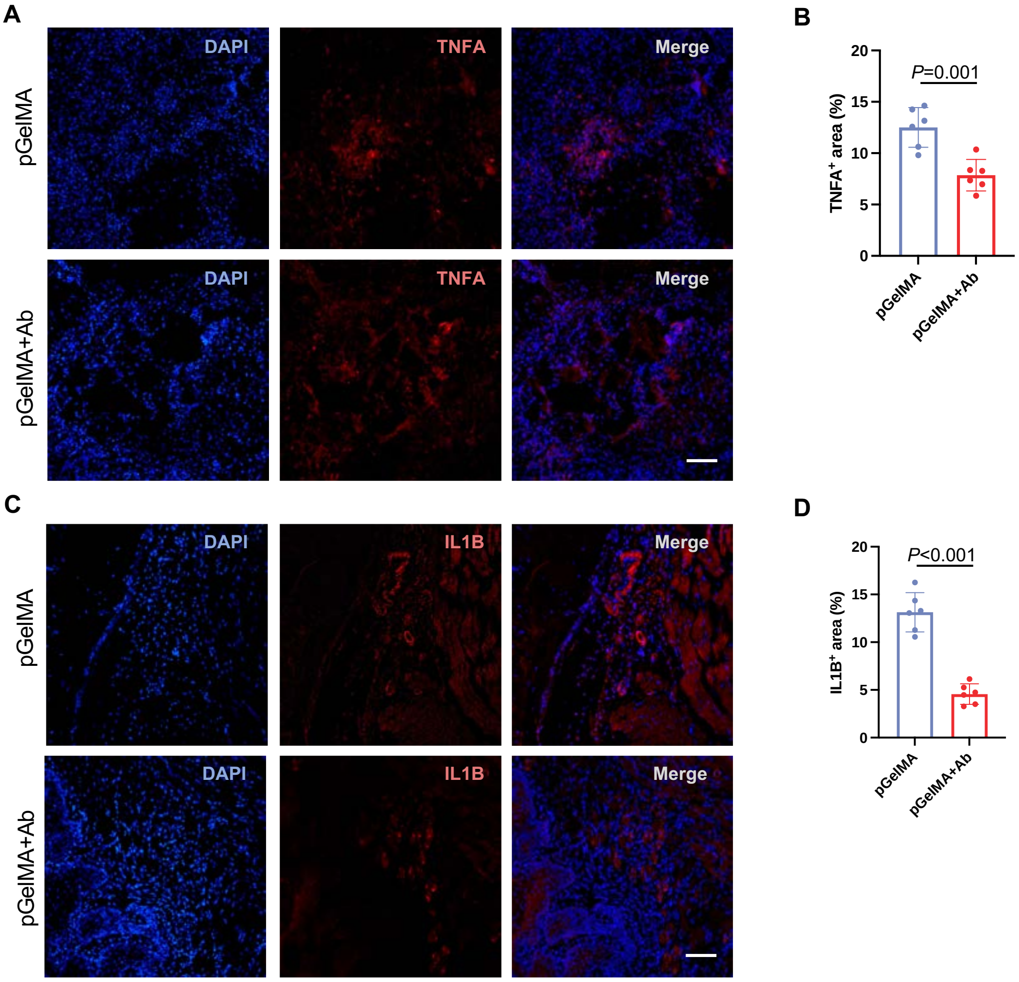

Supplementary Figure 15. Immunofluorescence staining of inflammatory factors. (**A, B**) Immunofluorescence staining and statistical analysis of TNFA after topical application of drug-loaded hydrogel. (**C, D**) Immunofluorescence staining and statistical analysis of IL1B after topical application of drug-loaded hydrogel.
